# Construction of A GBS-Based High-Density Genetic Map and Flower Color-Related Loci Mapping in Grasspea (*Lathyrus sativus* L.)

**DOI:** 10.3390/plants11162172

**Published:** 2022-08-21

**Authors:** Xiaopeng Hao, Tao Yang, Yan Wang, Rong Liu, Xue Dong, Jiandong Zhao, Jucai Han, Xuxiao Zong, Jianwu Chang, Huiping Liu

**Affiliations:** 1 College of Plant Protection, Shanxi Agricultural University, Taigu 030801, China; 2 Center for Agricultural Genetic Resources Research, Shanxi Agricultural University, Taiyuan 030031, China; 3 Key Laboratory of Crop Gene Resources and Germplasm Enhancement on Loess Plateau, Ministry of Agriculture, Taiyuan 030031, China; 4 Shanxi Key Laboratory of Genetic Resources and Genetic Improvement of Minor Crops, Taiyuan 030031, China; 5 Institute of Crop Sciences, Chinese Academy of Agricultural Sciences (CAAS), Beijing 100089, China

**Keywords:** genetic linkage map, genotyping-by-sequencing (GBS), *Lathyrus sativus* L. (grasspea), SNP, QTLs

## Abstract

Grasspea (*Lathyrus sativus* L.), a legume crop with excellent resistance to a broad array of environmental stressors, has, to this point, been poorly genetically characterized. High-density genetic linkage maps are critical for draft genome assembly, quantitative trait loci (QTLs) analysis, and gene mining. The lack of a high-density genetic linkage map has limited both genomic studies and selective breeding in grasspea. Here, we developed a high-density genetic linkage map of grasspea using genotyping-by-sequencing (GBS) to sequence 154 grasspea plants, comprising 2 parents and 152 F_2_ progeny. In all, 307.74 Gb of data was produced, including 2,108,910,938 paired-end reads, as well as 3536 SNPs mapped to seven linkage groups (LG1–LG7). With an average length of 996.52 cM per LG, the overall genetic distance was 6975.68 cM. Both the χ^2^ test and QTL analysis, based on the Kruskal–Wallis (KW) test and interval mapping (IM) analysis, revealed the monogenic inheritance of flower color in grasspea, with the responsible QTL located between 308.437 cM and 311.346 cM in LG4. The results can aid grasspea genome assembly and accelerate the selective breeding of new grasspea germplasm resources.

## 1. Introduction

The grasspea (*Lathyrus sativus* L.) is an annual, cool-season legume with an ~8.2 Gb diploid genome (2*n* = 14) [1,2,3,4]. Grasspea originated in the Eastern Mediterranean and was domesticated in the Balkans about 8000 BC [5]. Today, grasspea is considered an orphan crop due to its limited cultivation area. However, it is a hardy and climate-smart crop that can be cultivated in environments which are unsuitable for the cultivation of other crops [6,7,8,9]. Grasspea is highly tolerant of an array of abiotic stressors, including drought, flooding, nutrient scarcity, and high soil salinity and alkalinity, and shows good resistance to several plant pathogens, including rust and powdery mildew [10,11,12,13,14,15]. Like other legumes, grasspea has the ability to fix nitrogen, thereby potentially reducing the need for exogenous fertilizer application. Because of these characteristics, grasspea has the potential to meet the food demands of low-income countries and others with marginal land [16]. Unfortunately, grasspea contains the neurotoxin *N*-oxalyl-L-α,β-diaminopropionic acid (β-ODAP), which causes neurolathyrism when consumed in large quantities [17,18,19]. Despite this limitation, as of 2014, the grasspea planting area reached 1,500,000 hectares, with an annual production of 1,200,000 tons [20]. Even with moderate acreage and productivity, grasspea is gaining increasing attention due to its resilience under unfavorable environmental conditions [21].

Genetic linkage mapping is vital for genome assembly and trait loci mapping. Since the first genetic linkage map was published in 1913, a plethora of linkage maps based on phenotypic, protein, and DNA markers have been produced [22]. Due to the limitations of phenotypic and protein markers, DNA markers are increasingly utilized for the generation of genetic linkage maps [23]. In 1980, restriction fragment length polymorphism (RFLP) markers were used to construct the first DNA-based human genetic map [24]. More recently, the advancement of next-generation sequencing (NGS) technology has accelerated the development and application of molecular markers for use in constructing genetic maps, including genotyping-by-sequencing (GBS), restriction-site-associated DNA sequencing (RAD-seq), specific-locus amplified fragment sequencing (SLAF-seq), and re-sequencing [25,26,27,28]. Of these, GBS is considered one of the most efficient and cost-effective technologies for creating linkage maps. In comparison to the other genotypeing methods, GBS is more precise, efficient, and cost-effective [29]. However, despite its potential to address food insecurity, no major genomic evaluation has been conducted in grasspea. Because of its poor yield, large genome, high outcrossing rate, and neurotoxic disadvantage, grasspea has received little attention from researchers and, as a result, grasspea molecular biology and genomic research has fallen significantly behind that of more prominent legumes [16,18,30].

In recent years, smart breeding methods based on marker-assisted selection (MAS) have been used for crop breeding, and a genetic linkage map is a prerequisite for this technique. It is now possible to create a high-density genetic map for the grasspea [31]. Chowdhury (1999) developed the first grasspea genetic linkage map based on 69 markers produced from an F_2_ population of 102 parents and progeny, including 65 random amplified polymorphic DNA (RAPD) markers, 3 isozyme markers, and 1 phenotypic marker, mapped to 14 linkage groups (LGs) [32]. Next, Skiba (2004) developed a genetic map using backcross individuals produced from parents either resistant or susceptible to Ascochyta Blight induced by *Mycosphaerella pinodes* [33]. Using 47 RAPD markers, 7 sequence-tagged microsatellite sites (STMS) markers, and 13 sequence-tagged sites (STS)/cleaved amplified polymorphic sequence (CAPS) markers, the map covered a genetic distance of 803.1 cM, with an average of 15.8 cM per marker, mapped to nine LGs [33]. Most recently, Diversity Array Technology sequencing (DArTseq) has been used to create a true high-density genetic map using 105 recombinant inbred lines (RILs) and 2149 markers, including 1472 SilicoDArT markers and 677 single-nucleotide polymorphisms (SNPs) [34]. Based on RIL screening, *LsMLO1*, a key gene responsible for powdery mildew susceptibility in both *L. sativus* and *L. cicera*, was identified, described, and mapped to the upper portion of LG1, at position 18.246 cM, in the *L. sativus* genetic map [34]. As the closest wild relative of *L. sativus*, it is perhaps equally important to examine the genome of *L. cicera.* To that end, Santos (2018, 2020) created two genetic maps for *L. cicera*, including 307 and 1468 markers, respectively, mapped to nine LGs [35,36].

Phenotypic markers, such as flower, seed, and fruit color and leaf form, are critical for differentiating between various germplasm resources [37,38,39]. In grasspea, flower color shows the greatest phenotypic diversity, with accessions exhibiting blue, white, or pink flowers. This diversity may be related to functional differences in pollinator attraction and success [40]. In this work, artificial hybridization between two grasspea accessions, IF1347 and K714, was undertaken in order to develop new, more diverse germplasm resources. An F_2_ population of 152 individuals was obtained and used to produce a high-density genetic map, resulting in the discovery of several flower-color-associated loci. It is our hope that these findings can aid in the assembly of a draft grasspea genome and enable future selective breeding efforts for this promising legume crop.

## 2. Results

### 2.1. GBS Data Analysis and Assessment

A total of 154 samples, including 2 parents and 152 F_2_ progeny, were sequenced, yielding 307.77 Gb of raw data. After quality control, 307.74 Gb of high-quality reads, including 2,108,910,938 paired-end reads, were available for SNP calling. The raw data contained an average of 99.97% clean reads, with a Q20 of 95.24%, a Q30 of 87.66%, and GC of 38.6%. The male (P1, 2,272,102,272 bp) and female (P2, 2,300,245,632 bp) parents were sequenced and the raw data contained an average of 100% clean reads (Table S1). For the 152 F_2_ progeny, clean reads varied from 1,301,804,352 to 2,670,197,184 bp, with an average of 1,994,496,457 bp.

### 2.2. SNP Calling and Genotyping

Clean reads were aligned using the ‘Rbp’ reference genome, based on the European grasspea accession ‘LS007’, supplied by JIC [7] (Table S2). A total of 15,617,673 clean reads were mapped for the male parent and 15,808,872 clean reads were mapped for the female parent. An average of 13,667,660 clean reads were mapped to the draft genome for the 152 F_2_ progeny. In all, approximately 98.68% of the data could be mapped to the ‘Rbp’ genome. Only the clean reads mapped to the draft genome were used for SNP calling and genotyping.

**Figure 1:**
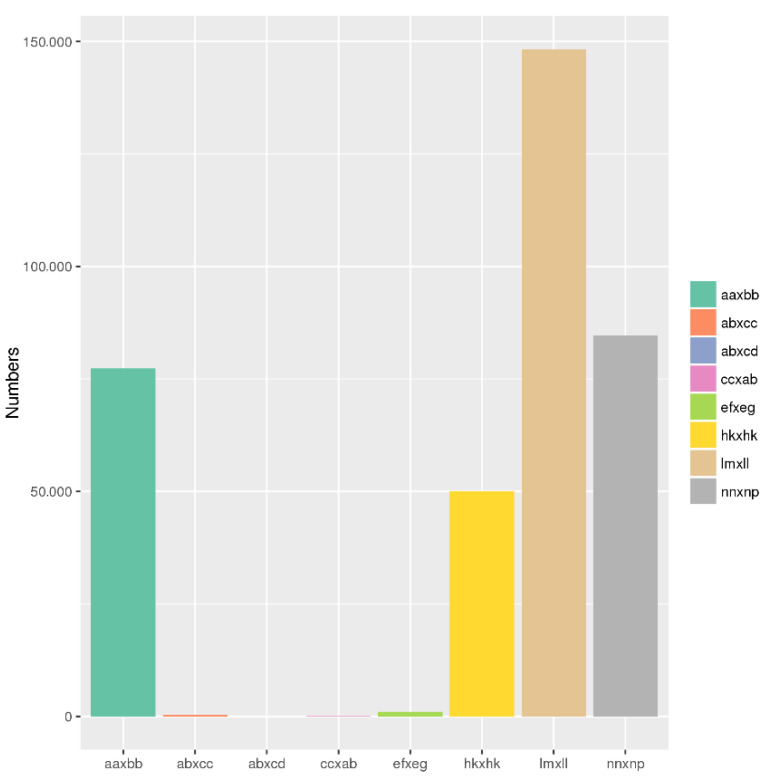
Frequency of polymorphism segregation types between K714 and IF1347. Different colors (from left to right) represent the 7 segregation types.

The clean reads of both parents and F_2_ progeny were aligned to the ‘Rbp’ draft reference genome using the Burrows-Wheeler Aligner (BWA) software with the setting “mem -t 4 -k 32 -M -R” [41]. SAMtools was used to call SNPs in all samples once the alignment files (SAM) were transformed into BAM files [42]. For the male parent (P1), 551,846 SNPs were called, with 39.06% (215,557) homozygous and 60.94% heterozygous SNPs (Table S3). For the female parent (P2), 522,270 SNPs were called, with 61.19% (319,583) homozygous and 38.81% heterozygous SNPs (Table S3). An average of 244,236 homozygous SNPs were called across F_2_ progeny, ranging from 38.42% to 55.95% in each F_2_ individual. Polymorphic SNPs in P1 and P2 were categorized into seven segregation patterns, including ‘ef × eg’, ‘hk × hk’, ‘lm × ll’, ‘nn × np’, ‘aa × bb’, ‘ab × cc’, and ‘cc × ab‘. However, only markers with ‘aa × bb’-type segregation, encompassing 77,346 SNPs between the parents, were chosen for genetic mapping of the F_2_ population (Figure 1).

### 2.3. Genetic Linkage Map Constructing and Verification

For the F_2_ progeny, abnormal markers with <70% accuracy were eliminated from further analyses. To identify segregation distortion, chi-square (χ^2^) tests were performed on all SNPs. Markers exhibiting a statistically significant χ^2^ result (*p* < 0.001) or aberrant bases were eliminated. Finally, 4428 markers with ‘aa × bb’-type segregation were kept. A high-resolution genetic linkage map was created using JoinMap4.1, based on the Kosambi genetic distance calculation and regression ordering method. A total of 3536 SNP markers were mapped to seven LGs, with a total genetic length of 6975.68 cM and an average LG length of 996.52 cM, ranging from 504.11 cM (LG1) to 1367.47 cM (LG4). The average length between two neighboring markers was 2.21 cM. The number of anchoring markers per LG ranged from 290 (LG4) to 1240 (LG1), with an average of 505 markers per LG (Supplementary file S1). LG1 had the highest marker density (1.1 cM per interval), while LG7 had the lowest marker density (2.98 cM per interval). The majority of the gaps between markers were less than 5 cM in length, with the largest gap consisting of 31.47 cM between mk1930 and mk4366 in LG4. In total, 3205 SNPs (<5 cM), accounting for 90.64% of 3536, were mapped to LG1-LG7 (Table 1 and Table 2, and Figure S1).

Both haplotype map and heat map analysis are crucial for evaluating the quality of high-resolution genetic maps. Haplotype mapping can identify markers with genotyping errors in the segregating population and heat mapping can visualize linkage connections per marker in each LG. These two methods were employed to discover any possible ordering errors or linkage connections between markers within a single LG across the seven *L. sativus* LGs. Haplotype maps were constructed using genotypes of SNP markers from both parents and F_2_ progeny. The recombination connection between markers in the haplotype map was utilized to detect marker ordering mistakes, which were indicated by double crossings (Figure S2). Between both parents and F_2_ progeny, the majority of areas across the seven haplotype maps exhibited similar color, indicating clear recombination blocks and ensuring the creation of a high-quality genetic map. Based on the heat maps of the seven LGs, we observed a significant connection between two nearby markers in seven LGs. Specifically, as the genetic distance between two markers increased, the link between them decreased, revealing the correct sequence of markers in the seven LGs (Figure S3).

### 2.4. QTL Analysis of Flower Color-Related Traits

**Figure 2:**
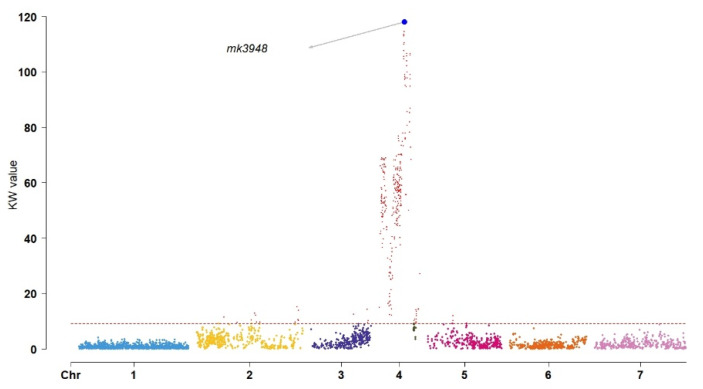
Kruskal–Wallis (KW) analysis (*p* < 0.01). The grey arrow and blue point indicate the most significant value. Chromosomes (left to right, from 1 to 7) are represented with different colors along the *x*-axis.

**Figure 3:**
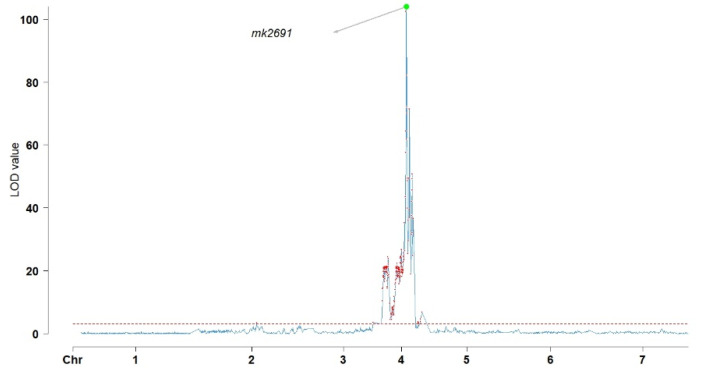
Interval mapping (IM) test (LOD > 3.0). The grey arrow and green point indicate the most significant value. Chromosomes (left to right) are represented from 1 to 7 along the *x*-axis.

Individuals with blue flowers (male) outnumbered those with white flowers (female) by a ratio of 115:37, in agreement with Mendel’s idealized 3:1 segregation ratio, demonstrating monogenic inheritance (χ^2^ = 0.0175, *p* > 0.05). Kruskal–Wallis (KW) analysis and interval mapping (IM) were employed to identify the significant loci affecting flower color in the F_2_ population. According to the KW analysis, the QTL regulating flower color was found in LG4 at location 308.437 cM (mk3948) and had a KW value of 117.99 (Figure 2). IM similarly identified the genes in LG4, but with a location of 311.346 cM (mk2691), a LOD value of 103.98, and a percentage of the variance explained (PVE) of 96.5% (Figure 3).

## 3. Discussion

Grasspea, an orphan food crop, shows strong resilience in the face of difficult environmental conditions, including drought, flooding, high salinity, and cold temperatures. Although grasspea has great potential to be developed into a robust crop for challenging environments, genetic research has so far been lacking [1,7,16,30]. Genetic linkage mapping is an essential tool for assembling genomes and gene mining. Previously, RAPD, simple sequence repeat (SSR), SNP, and DArT markers have been used to create grasspea genetic maps using PCR, transcriptomic, and GBS technologies [32,33,34,35,36]. Based on NGS techniques, GBS is an efficient and powerful method for identifying enrichment variation [43]. Through DNA extraction, restriction enzyme digestion, fragment selection, library assembly, and bioinformatics analysis, many variants, including SNPs and insertions/deletions (InDels), may be identified [44,45]. After the most highly significant genes are revealed, GBS may be used for high-density genetic mapping, genetic diversity and population structure analysis, QTL mapping, and genome-wide association studies (GWAS) [46,47,48]. For cool-season legumes, particularly peas, lentils, chickpeas, fava beans, and grasspeas, with large genomes (~1.0–13.0 Gb), GBS has significant benefits over *de novo* sequencing and re-sequencing in terms of speed, efficiency, and labor- and cost-effectiveness. In this study, GBS captured 307.74 Gb of clean data comprising 2,108,910,938 high-quality paired-end reads, with a Q30 of 87.66%. Clean reads were mapped to the ‘Rbp’ draft grasspea genome, ultimately resulting in 77,346 SNPs with an ‘aa × bb’ segregation pattern (other patterns were excluded from further analysis). Of these, 3536 SNPs were mapped to the seven LGs, resulting in a total genomic length of 6975.68 cM with an average genomic marker interval of 2.21 cM. This genetic map can be used to assist in locating continuous sequences (contigs) on the physical map for *L. sativus* draft genome assembly.

High-resolution genetic maps are critical for modern selective breeding. The use of such maps has led to the identification of a large number of agronomically relevant QTLs in a wide array of food crops, including maize, foxtail millet, and jujube [49,50,51]. Recently, high-resolution genetic maps have been created for both *L. sativus* and the closely related *L. cicera* [34,36]. In the most recent of these maps, 1468 markers were identified across nine LGs (seven main LGs and two minor LGs), with a total length of 712.35 cM and an average interval distance of 0.65 cM. These markers included 730 silicoDArT markers, 623 SNPs, 110 expressed sequence tag-derived SSR (ESSR) markers, and 5 intron targeted amplified polymorphism (ITAP) markers. Three *Erysiphe pisi*–responsive and one *Erysiphe trifolii*-responsive QTL were found to explain 13.0%, 10.6%, 8.2%, and 16% of the phenotypic variation, respectively [36]. More recently, utilizing on DArT-Seq, 2149 markers were mapped to 10 LGs (7 main LGs and 3 minor LGs), with a total genetic distance of 674.4 cM and an average interval distance of 0.41 cM [34]. The SNP related to *LsMLO1*, controlling powdery mildew susceptibility, was found on LG1 at position 18.246 cM, and a transversion (G > T) is likely responsible for phenotypic variation in powdery mildew resistance [33]. In this work, the constructed genetic map covered a total genetic distance of 6975.68 cM with an average interval distance of 2.21 cM, which is three–five times larger than previous reported for *L. sativus* or *L. cicera* [27,28,31,34,36,50]. Due to a high outcrossing rate, our parental accessions exhibited high heterogeneity, only containing between 38.81% and 60.94% heterozygous SNPs, resulting in longer marker interval distances. The purification of parental germplasm may be necessary to develop a high-quality high-resolution genetic linkage map with increased SNP density and decreased marker interval distances. Additionally, we found that more than 1/3 of all SNPs (1240) were mapped to LG1, which contained 2.6–4.3 times more SNPs than the other LGs. As mentioned above, previous high-resolution genetic maps of *L. sativus* and *L. cicera* contained two or three minor LGs alongside to the seven primary LGs. This may be due to chromosomal rearrangement, as chromosomal instability has been reported in grasspea since 1982 [52].

Flower color is an important reproductive characteristic in diverse plant species, with both monogenic or polygenic inheritance [53,54]. We found that, in grasspea, flower color is controlled by a single gene through monogenic inheritance with a segregation ratio of 3:1 (χ^2^ = 0.0175, *p* > 0.05). This is similar to both cowpea and soybean, in that QTL analysis of these species revealed the presence of a single QTL controlling flower color [55,56]. Specifically, the gene *Vigun07g110700*, responsible for flavonoid biosynthesis, was identified as controlling flower color, and is similar to the Arabidopsis *TT8* gene, which is responsible for the biosynthesis of anthocyanins [55]. These results are in contrast to the unrelated species *Salvia miltiorrhiza*, in which two QTLs appear to be responsible for flower color (qfcRGB4 and qfcRGB5) [38]. These flower color QTLs were identified on LG4 with a KW value of 117.99 at position 308.437 cM (mk3948) and a LOD value of 103.98 at position 311.346 cM (mk2691, PVE = 96.5%), based on both KW and IM analysis, demonstrating the consistency of these two methods. Only flower size is more important than flower color in attracting bees and ensuring successful pollination [57,58].

## 4. Materials and Methods

### 4.1. Plant Materials

The F_1_ population was created through the artificial hybridization of two grasspea cultivars: the blue-flowered ICARDA accession ‘IF1347’ was used as the male parent (P1) and the white-flowered Polish accession ‘k714’ was used as the female parent (P2). The P1, P2, and F_2_ population from 2016 were planted at the experimental location (38.14° N, 113.04° E, altitude 1215 m), Nantou Village, Yu County, Shanxi Province, China.

### 4.2. DNA Sample Preparation

Young leaves from both parents and progeny were harvested four weeks after seeding in May 2018. The leaves were immediately frozen in liquid nitrogen and held at −80 °C in the laboratory. The genomic DNA (gDNA) from 154 grasspea plants (2 parents and 152 F_2_ progeny) was extracted using a DNAsecure Plant Kit (Tiangen Biotec Co., Ltd., Beijing, China). The quality of the gDNA was determined using 1% agarose gel electrophoresis. The purity and concentration of the gDNA were determined using a Nanophotometer spectrophotometer (Implen, Westlake, CA, USA) and a Fluorometer 2.0 (Life Technologies, Carlsbad, CA, USA).

### 4.3. Library Construction and GBS Sequencing

GBS sequencing was conducted according to the method of Elshire et al., 2011 [29], with minor improvements. Briefly, the gDNA from 154 grasspea plants (2 parents and 152 F_2_ progeny) was first digested by MseI (5’-T!TAA-3’) (New England Biolabs, ‘NEB’, Ipswich, MA, USA) at 37 °C, then subjected to restriction-ligation by T4 DNA ligase (NEB) and ATP (NEB) with a MseI Y-adapter *N*-containing barcode at 65 °C, followed by a second digestion with HaeIII (5’-GG!CC-3’) (NEB) at 37 °C. The digested fragments were purified using Agencourt AMPure XP (Beckman Coulter, ‘Beckman’, Brea, CA, USA) and used for PCR amplification, utilizing Phusion Master Mix (NEB) to add universal and index primers, as well as i5 and i7 index sequences, to the digested fragments. The purified PCR fragments (425–490 bp including indexes and adaptors) were screened and extracted using a 2% agarose gel with the Gel Extraction Kit (Qiagen, Valencia, CA, USA). The amplification products were purified using Agencourt AMPure XP (Beckman) and diluted before sequencing. Finally, an Illumina NovaSeq 6000 platform (Illumina, San Diego, CA, USA) was used to perform 150-bp paired-end sequencing by Tianjin Novogene Technology Co., Ltd. (Tianjin, China).

### 4.4. Sequencing Data Analysis and Quality Control

The barcodes in the raw data (reads) were used to classify the sequencing data from both parents and progeny. Because low-quality paired-end reads, caused primarily by base-calling duplicates and adapter contamination, can lead to unreliable raw data, the raw data (in FASTQ format) was subjected to quality control (QC) using C and Perl scripts. The QC criteria were as follows: (1) filtering read pairs with sequencing adapter; (2) filtering read pairs with one read comprising N > 10%; (3) filtering read pairs with >50% low-quality bases (Q ≤ 5).

### 4.5. SNP Identification and Genotyping

The BWA software was used to map the clean data from each sample to the reference draft genome (settings: mem -t 4 -k 32 -M -R) [41]. The SAMtools program was used to convert alignment data to BAM files [41]. Polymorphic markers between P1 and P2 were identified and classified into 7 segregation patterns (ef × eg, hk × hk, lm × ll, nn × np, aa × bb, ab × cc, and cc × ab) using the JoinMap 4.1 software [59]. Only markers with ‘aa × bb’-type segregation were chosen for genetic mapping of the F_2_ population.

### 4.6. Genetic Linkage Map Construction and Evaluation

All SNPs with discordant segregation ratios (*p* < 0.001), lacking ≥ 30% of the genotype data, or containing aberrant bases were removed using an in-house Perl script. JoinMap 4.1 software was used to calculate genetic distance using the Kosambi function, and the regression mapping approach, with a LOD value of 2–30, was used to arrange the markers into 7 LGs [60]. Finally, JoinMap 4.1 was used to identify marker order in each LG, and a Perl script SVG was used to visualize output maps. Both haplotype and heat maps were generated to analyze the genetic maps using R 4.1.0.

### 4.7. Flower Color Data Collection and QTL Analysis

At flowering time, the flower color of both grasspea parents and F_2_ progeny was examined. All progeny exhibited either blue or white, in accordance with the flower color of the male (blue) and female (white) parent. MapQTL 5.0 and CMplot by R 4.1.0 were used for the qualitative trait analysis and Manhattan figure drawing. χ^2^ tests, KW tests, and IM analyses were used to identify monogenic and oligogenic features, with a LOD > 3.0 indicating statistical significance.

## 5. Conclusions

An F_2_ population consisting of 152 offspring, along with the 2 parents, was sequenced by GBS and used to create a high-density genetic linkage map for grasspea. In all, a total of 3536 SNP markers were mapped to seven LGs, with a total genetic distance of 6975.68 cM and an average genetic distance of 2.21 cM. The phenotypic segregation ratio analysis (χ^2^ test) of F_2_ flower color revealed that this trait is governed by monogenic inheritance. The grasspea flower-color-related QTLs were located on LG4 by both KW and IM analysis. These results provide an important resource for the accelerated development of improved grasspea germplasm.

## Figures and Tables

**Table 1:** Grasspea genetic linkage map statistical information.

LG ^a^	Number of SNPs	Genetic Distance (cM)/LG	Avg. Genetic Distance (cM)/LG	Max. Gap (cM)/LG
LG1	1240	1367.47	1.1	10.41
LG2	476	1328.02	2.79	29.15
LG3	354	751.70	2.12	18.29
LG4	290	504.11	1.74	31.47
LG5	367	924.76	2.52	29.16
LG6	427	959.59	2.25	18.42
LG7	382	1140.03	2.98	25.22
Total	3536	6975.68	2.21	31.47

a: grasspea linkage group (LG).

**Table 2:** Statistics regarding the gap between markers across the grasspea genetic linkage map.

LG ^a^	Number of SNPs in <5 cM	Number of SNPs in 5–10 cM	Number of SNPs in 10–20 cM	Number of SNPs in >20 cM	Number of SNPs in <5 cM Ratio (%)
LG1	1228	11	1	0	99.03
LG2	399	51	22	4	83.82
LG3	316	25	13	0	89.27
LG4	272	11	5	2	93.79
LG5	306	39	20	2	83.38
LG6	380	35	12	0	88.99
LG7	304	60	17	1	79.58
Total	3205	232	90	9	90.64

a: grasspea linkage group (LG).

## Data Availability

The data presented in this study are available in http://db.cngb.org/cnsa/project/CNP0003158_ec6a3961/reviewlink/ (Project accession: CNP0003158), which can be accessed on 1 January 2023.

## Supplementary Materials

The following supporting information can be downloaded at: https://www.mdpi.com/article/10.3390/plants11162172/s1, Table S1. Statistic of sequencing data by GBS in male (P1), female (P2), and F_2_ individuals of grasspea. Table S2. Statistic of clean reads, mapped reads, and sequencing depth in male (P1), female (P2), and F_2_ individuals of grasspea. Table S3. Statistic of homozygous SNP, heterozygous SNP, and Homozygous SNP rate in male (P1), female (P2), and F_2_ individuals of grasspea. Figure S1. Genetic linkage map and SNP distribution across 7 LGs. The *x*-axis indicates the linkage group, and the *y*-axis indicates genetic distance of markers. Figure S2 (1–7). Similar source distribution of each marker from 1 to 7 LGs. The *x*-axis indicates the F_2_ individuals and the *y*-axis indicates the mapped SNPs. The ‘green (a)’ indicates genotype ‘aa’, ‘blue green (b)’ indicates ‘bb’, ‘purple (h)’ indicates ‘ab’, and ‘orange (-)’ indicates SNP absent. Figure S3 (1–7). Heat map of linkage relationships between adjacent markers from 1 to 7 LGs. The *x*-axis and *y*-axis indicate mapped SNPs, and ‘red’ to ‘yellow’ showed that linkage relationships (genetic distance) were from far to near. Supplementary file S1. Markers name, genetic distances, positions and genotyping of 3536 SNPs in 7 LGs.
