# Supplementary material for: Construction of A GBS-Based High-Density Genetic Map and Flower Color-Related Loci Mapping in Grasspea (Lathyrus sativus L.)
Source: Plants (Basel). 2022 Aug 21;11(16):2172. doi: 10.3390/plants11162172 (PMC9414002; doi:10.3390/plants11162172)
Supplement: Supplementary file 1 [file plants-11-02172-s001.zip › Table S1.pdf]

Table S1. Statistic of sequencing data by GBS in male (P1), female (P2), and F_2 individuals of grasspea.

Sample	Raw base(bp)	Clean base(bp)	Effective Rate(%)	Error Rate(%)	Q20(%)	Q30(%)	GC Content(%)
P1_K714	2272102272	2272102272	100	0.03	95.57	87.47	39.19
P2_IF1347	2300245632	2300245632	100	0.03	97.68	92.56	39.45
F2_1	2283011136	2282965344	100	0.03	96.62	90.4	38.9
F2_2	2241830304	2241780768	100	0.03	96.03	89.01	38.55
F2_3	2361699360	2361649824	100	0.04	95.94	88.85	38.58
F2_4	2547739296	2547679104	100	0.03	96.91	91.1	38.73
F2_5	2501559360	2501508960	100	0.03	96.31	89.7	39.08
F2_7	2208547872	2208508992	100	0.04	94.59	85.88	38.56
F2_8	2467122912	2467068192	100	0.04	95.13	87.05	39
F2_10	2670241248	2670197184	100	0.04	95.88	88.66	38.75
F2_11	2259451872	2259414144	100	0.04	94.59	85.86	39.11
F2_12	2535976224	2535926688	100	0.03	96.87	90.97	38.83
F2_13	2120599296	2120556960	100	0.03	97.13	91.56	38.87
F2_14	2507743008	2507695776	100	0.03	95.96	88.75	39.24
F2_15	2424794112	2424743712	100	0.03	96.08	89.16	38.98
F2_16	2019251808	2019213504	100	0.04	95.11	86.96	39.03
F2_17	2655298368	2655246528	100	0.04	94.61	85.84	38.6
F2_18	2458438560	2458386144	100	0.04	95.8	88.45	38.24
F2_19	2233653120	2233617408	100	0.04	94.91	86.54	38.88
F2_20	2232893952	2232852480	100	0.04	92.91	82.39	38.55
F2_22	2209794912	2209751136	100	0.03	96.14	89.2	38.43
F2_23	2404800000	2404747584	100	0.03	96.15	89.22	38.48
F2_25	2517990912	2517227136	99.97	0.04	93.64	84.66	37.34
F2_27	2259990144	2259296352	99.97	0.04	93.66	84.69	38.61
F2_28	2570560128	2569817952	99.97	0.04	93.23	83.89	38.97
F2_30	2451600000	2450885184	99.97	0.04	94.23	85.71	37.05
F2_31	2140070976	2139444288	99.97	0.04	94.11	85.55	38.06
F2_32	1796466816	1795940064	99.97	0.04	93.62	84.64	38.92
F2_33	1954895328	1954382976	99.97	0.04	92.37	82.15	39.04
F2_34	2050641792	2050022592	99.97	0.04	95.23	87.7	38.75
F2_35	2513643552	2512878912	99.97	0.04	94	85.33	38.4
F2_36	2216740032	2216075328	99.97	0.04	94.6	86.58	38.67
F2_39	2140122528	2139492096	99.97	0.04	94.16	85.68	38.72
F2_40	2061904320	2061311328	99.97	0.04	93.44	84.1	36.97
F2_41	2130170112	2129571936	99.97	0.04	93.15	83.56	38
F2_42	1833982272	1833499296	99.97	0.04	92.64	82.65	39.21
F2_43	1947126528	1946594880	99.97	0.04	92.59	82.55	39.02
F2_44	2225592576	2224957248	99.97	0.04	93.66	84.75	38.81
F2_45	1957419648	1956806784	99.97	0.04	94.6	86.67	38.83
F2_46	2173006656	2172353472	99.97	0.04	93.76	84.94	38.5
F2_47	2436010272	2435293152	99.97	0.04	93.66	84.78	38.42
F2_49	2133630144	2132996256	99.97	0.04	95.09	87.61	38.66

F2_50	2222868672	2222187552	99.97	0.04	94.24	85.91	38.97
F2_51	1795826880	1795351104	99.97	0.04	91.77	81.23	38.84
F2_52	1841613408	1841086080	99.97	0.04	92.69	82.91	39.22
F2_53	1843815744	1843262784	99.97	0.04	93.63	84.7	39
F2_54	1676830752	1676830752	100	0.03	95.22	87.84	37.59
F2_55	1600013952	1599984288	100	0.03	95.33	87.97	38.45
F2_56	1680229440	1680196032	100	0.03	94.87	87.17	38.82
F2_58	1917863712	1917863712	100	0.03	95.61	88.59	37.95
F2_59	1613314368	1613314368	100	0.03	95.47	88.38	38.17
F2_60	1636356096	1636328448	100	0.03	95.25	87.91	38.87
F2_61	1416315744	1416295296	100	0.04	94.69	86.61	38.93
F2_62	1836969696	1836969696	100	0.03	96.2	89.82	38.64
F2_63	1728237312	1728211968	100	0.03	95.52	88.41	38.17
F2_64	1763581248	1763549568	100	0.03	95.72	88.92	38.53
F2_65	1563604416	1563579360	100	0.03	95.62	88.62	38.51
F2_66	1301804352	1301804352	100	0.03	95.42	87.97	36.99
F2_67	1677996288	1677964896	100	0.03	94.77	86.84	37.89
F2_69	1549139904	1549107936	100	0.03	94.84	86.85	38.93
F2_70	1542142080	1542142080	100	0.04	94.81	86.81	38.92
F2_71	1555544160	1555517376	100	0.03	95.21	87.83	38.7
F2_72	1978591392	1978096608	99.97	0.04	94.94	87	38.8
F2_73	1848368160	1847922624	99.98	0.04	93.84	84.75	38.8
F2_74	1842972768	1842522624	99.98	0.03	96.1	89.38	38.77
F2_75	2059958016	2059457760	99.98	0.04	95.26	87.63	38.41
F2_76	1884204576	1883739456	99.98	0.04	95.7	88.64	38.72
F2_78	1897443360	1896998688	99.98	0.04	95.4	87.95	38.59
F2_80	2231352864	2230803360	99.98	0.04	94.75	86.51	37.23
F2_82	2402142624	2401584192	99.98	0.04	94.58	86.11	37.79
F2_83	1807746624	1807306848	99.98	0.04	94.21	85.5	38.99
F2_86	1676104992	1675688256	99.98	0.04	94.14	85.34	39.11
F2_87	1705316544	1704895200	99.98	0.04	95.04	87.23	38.87
F2_89	1886926176	1886463648	99.98	0.04	95.71	88.7	38.74
F2_91	1863915264	1863447840	99.97	0.04	95.06	87.3	38.45
F2_92	1819883520	1819434816	99.98	0.04	95	87.19	38.39
F2_94	1676025792	1675617696	99.98	0.03	96.07	89.47	38.56
F2_95	1919332512	1918859904	99.98	0.04	95.41	88.05	38.73
F2_96	1731451392	1731042720	99.98	0.04	93.51	84.19	38.34
F2_98	1620661536	1620292896	99.98	0.04	94.19	85.49	38.84
F2_99	1636348320	1635952608	99.98	0.04	94.96	87.1	38.83
F2_100	1867213440	1866793536	99.98	0.04	93.62	84.34	39.03
F2_101	1784416320	1783976256	99.98	0.03	96	89.3	38.66
F2_103	1626507936	1626114816	99.98	0.03	96.28	89.9	38.81
F2_105	1856414304	1855977984	99.98	0.04	95.08	87.24	38.96
F2_106	1810973952	1810529568	99.98	0.04	95.19	87.59	38.86
F2_108	1691731008	1691324064	99.98	0.04	94.16	85.41	38.95
F2_109	1482608448	1482264000	99.98	0.04	93.59	84.23	38.34

F2_110	2218140000	2217596832	99.98	0.04	94.84	86.78	37.47
F2_111	1700862048	1700479584	99.98	0.04	93.96	85.03	38.87
F2_114	1665249984	1664884224	99.98	0.04	91.92	81.03	38.5
F2_115	1909200384	1908748800	99.98	0.04	95.22	87.53	38.38
F2_117	1833009408	1832562432	99.98	0.04	95.24	87.61	38.5
F2_122	2589269760	2589217344	100	0.04	95.97	88.82	38.07
F2_123	2231254080	2231212608	100	0.04	95.9	88.68	38.91
F2_124	2069445600	2069414208	100	0.04	95.56	87.94	39.16
F2_126	2264635296	2264587488	100	0.03	96.32	89.59	38.48
F2_127	2369380320	2369338272	100	0.03	96.22	89.41	38.44
F2_128	1933696512	1933661088	100	0.04	95.9	88.69	39.14
F2_130	1859516928	1859482368	100	0.04	94.88	86.41	39.12
F2_131	1852167744	1852133184	100	0.03	96.94	91.02	38.84
F2_133	2142859680	2142809856	100	0.03	96.15	89.22	38.57
F2_137	1969933824	1969454592	99.98	0.04	94.97	87.06	38.56
F2_138	2070793728	2070744192	100	0.03	96.57	90.27	38.99
F2_141	2008816704	2008778112	100	0.03	96.32	89.65	38.89
F2_143	2148610176	2148573888	100	0.04	95.71	88.13	37.67
F2_144	2008632096	2008591200	100	0.04	95.4	87.51	38.81
F2_145	2052496800	2052461664	100	0.04	95.19	87.07	39.11
F2_146	1799096256	1799066880	100	0.04	95.08	86.88	39.3
F2_147	1579562496	1579562496	100	0.03	95.63	88.89	38.59
F2_149	1578846528	1578826368	100	0.03	95.3	88.08	38.37
F2_150	1724923584	1724895360	100	0.03	95.15	87.83	38.3
F2_151	1897329312	1897297632	100	0.03	95.91	89.44	38.3
F2_152	1787416128	1787384448	100	0.03	95.42	88.38	38.55
F2_153	1742894496	1742867424	100	0.04	94.43	86.17	38.38
F2_156	1629970848	1629970848	100	0.03	94.67	86.75	38.8
F2_157	1747306944	1747306944	100	0.03	95.08	87.6	38.5
F2_158	1698477696	1698440256	100	0.04	94.21	85.76	38.82
F2_159	1752586560	1752552864	100	0.03	95.88	89.32	38.48
F2_160	1681602048	1681602048	100	0.03	96.13	89.83	38.5
F2_161	1663459776	1663421184	100	0.03	95.31	87.9	38.97
F2_162	1575136224	1575106272	100	0.03	95.21	87.92	38.7
F2_165	1524634560	1524634560	100	0.04	94.65	86.59	38.84
F2_166	1470161664	1470136320	100	0.04	94.25	85.84	38.6
F2_167	2181214080	2181168288	100	0.03	95.35	88.06	37.69
F2_169	2158576128	2158054848	99.98	0.04	95.01	87.14	37.73
F2_170	1807229664	1807229664	100	0.04	94.57	86.5	38.67
F2_173	1842442560	1842008256	99.98	0.04	95.44	87.97	37.81
F2_174	1776898656	1776898656	100	0.04	93.16	83.58	38.38
F2_176	1957796352	1957769280	100	0.03	95.4	88.22	37.59
F2_177	1852606080	1852579584	100	0.03	95.47	88.31	38.22
F2_178	1879891200	1879879104	100	0.03	96.59	90.1	37.55
F2_180	1877317632	1877305824	100	0.03	96.45	89.8	39.05
F2_182	2289349440	2289332160	100	0.03	96.38	89.62	39

F2_183	2546809056	2546790912	100	0.03	97.05	91.31	38.78
F2_186	2175363648	2175347808	100	0.03	97.54	92.63	38.78
F2_188	2221193376	2221178400	100	0.03	97.18	91.64	38.38
F2_190	2291917248	2291898816	100	0.03	97.06	91.41	38.52
F2_192	2055414528	2055394368	100	0.03	97.72	93.11	38.58
F2_194	2048434848	2048420160	100	0.03	97.31	92.05	39.05
F2_195	2435059872	2435043168	100	0.03	96.13	89.08	38.41
F2_196	1690063200	1690049088	100	0.03	96.38	89.74	38.98
F2_197	2269705824	2269688544	100	0.03	96.97	91.14	38.73
F2_198	1915636320	1915180128	99.98	0.04	94.61	86.33	38.98
F2_199	1987848000	1987832448	100	0.03	95.86	88.54	38.95
F2_200	2270493216	2270479968	100	0.03	97.68	92.99	38.83
F2_201	2277808416	2277791136	100	0.03	97.89	93.5	38.84
F2_202	2354699520	2354682816	100	0.03	96.97	91.06	38.98
F2_203	2127743424	2127729600	100	0.03	97.11	91.53	38.81
F2_205	2274279552	2274264288	100	0.03	96.38	89.68	38.99
F2_207	2042512416	2042000640	99.97	0.04	95.32	87.78	38.15
F2_211	2397913632	2397894048	100	0.03	96.02	88.81	38.31
Average	1998502801	1998284477	99.99	0.04	95.24	87.66	38.60
