# Supplementary material for: Construction of A GBS-Based High-Density Genetic Map and Flower Color-Related Loci Mapping in Grasspea (Lathyrus sativus L.)
Source: Plants (Basel). 2022 Aug 21;11(16):2172. doi: 10.3390/plants11162172 (PMC9414002; doi:10.3390/plants11162172)
Supplement: Supplementary file 1 [file plants-11-02172-s001.zip › Table S2.pdf]

Table S2. Statistic of clean reads, mapped reads, and sequencing depth in male (P1), female (P2), and F_2 individuals of grasspea.

Sample	Clean reads	Reads mapped on genome	Mapping rate(%)	Sequencing average depth[a]
P1_K714	15778488	15617673	98.98	13.52
P2_IF1347	15973928	15808872	98.97	13.88
F2_1	15853926	15669916	98.84	13.62
F2_2	15567922	15377596	98.78	14.04
F2_3	16400346	16201026	98.78	14.5
F2_4	17692216	17492857	98.87	14.6
F2_5	17371590	17154577	98.75	14.66
F2_7	15336868	15117484	98.57	13.95
F2_8	17132418	16937131	98.86	14.54
F2_10	18543036	18321807	98.81	15.17
F2_11	15690376	15485003	98.69	13.62
F2_12	17610602	17417301	98.9	14.69
F2_13	14726090	14568562	98.93	13.29
F2_14	17414554	17227506	98.93	14.38
F2_15	16838498	16641396	98.83	14.35
F2_16	14022316	13852005	98.79	13.13
F2_17	18439212	18207310	98.74	15.47
F2_18	17072126	16863106	98.78	15.12
F2_19	15511232	15325760	98.8	13.82
F2_20	15505920	15275973	98.52	14.45
F2_22	15345494	15159877	98.79	14.09
F2_23	16699636	16508508	98.86	14.99
F2_25	17480744	17181416	98.29	16.2
F2_27	15689558	15448373	98.46	13.88
F2_28	17845958	17558768	98.39	14.5
F2_30	17020036	16745380	98.39	16.26
F2_31	14857252	14623388	98.43	13.96
F2_32	12471806	12274211	98.42	12.31
F2_33	13572104	13354098	98.39	12.71
F2_34	14236268	14045420	98.66	12.95
F2_35	17450548	17185714	98.48	14.78
F2_36	15389412	15162359	98.52	13.63
F2_39	14857584	14635624	98.51	13.39
F2_40	14314662	14081016	98.37	15.02
F2_41	14788694	14553506	98.41	14.36
F2_42	12732634	12524207	98.36	12.42
F2_43	13518020	13306624	98.44	12.8
F2_44	15451092	15193744	98.33	13.56
F2_45	13588936	13376189	98.43	12.61

F2_46	15085788	14826367	98.28	13.6
F2_47	16911758	16632090	98.35	14.27
F2_49	14812474	14598728	98.56	13.37
F2_50	15431858	15201135	98.5	13.43
F2_51	12467716	12233184	98.12	12.4
F2_52	12785320	12581518	98.41	12.5
F2_53	12800436	12601461	98.45	12.47
F2_54	11644658	11474524	98.54	13.81
F2_55	11111002	10957692	98.62	12.46
F2_56	11668028	11497865	98.54	12.41
F2_58	13318498	13127462	98.57	14
F2_59	11203572	11047190	98.6	12.55
F2_60	11363392	11204976	98.61	12.26
F2_61	9835384	9705870	98.68	11.7
F2_62	12756734	12603941	98.8	12.9
F2_63	12001472	11843668	98.69	13.22
F2_64	12246872	12076117	98.61	12.76
F2_65	10858190	10723562	98.76	12.25
F2_66	9040308	8922074	98.69	13.63
F2_67	11652534	11486583	98.58	13.36
F2_69	10757694	10614324	98.67	12.04
F2_70	10709320	10576392	98.76	12.14
F2_71	10802204	10655076	98.64	12.21
F2_72	13736782	13556072	98.68	13.37
F2_73	12832796	12658163	98.64	12.76
F2_74	12795296	12642321	98.8	12.86
F2_75	14301790	14117909	98.71	13.84
F2_76	13081524	12918975	98.76	13.02
F2_78	13173602	13008761	98.75	13.18
F2_80	15491690	15281536	98.64	15.46
F2_82	16677668	16448215	98.62	16.17
F2_83	12550742	12375062	98.6	12.91
F2_86	11636724	11477090	98.63	12.27
F2_87	11839550	11680461	98.66	12.47
F2_89	13100442	12930203	98.7	13.02
F2_91	12940610	12762351	98.62	13.08
F2_92	12634964	12457145	98.59	13.06
F2_94	11636234	11489329	98.74	12.38
F2_95	13325416	13152619	98.7	13.02
F2_96	12021130	11842621	98.52	12.96
F2_98	11252034	11096809	98.62	12.38
F2_99	11360782	11209312	98.67	12.29
F2_100	12963844	12778667	98.57	12.84
F2_101	12388724	12231449	98.73	12.8
F2_103	11292464	11152143	98.76	12.15

F2_105	12888736	12729842	98.77	12.84
F2_106	12573122	12410099	98.7	12.81
F2_108	11745306	11577578	98.57	12.38
F2_109	10293500	10138376	98.49	12.42
F2_110	15399978	15186142	98.61	15.5
F2_111	11808886	11638940	98.56	12.48
F2_114	11561696	11375668	98.39	12.69
F2_115	13255200	13057345	98.51	13.22
F2_117	12726128	12559545	98.69	13.06
F2_122	17980676	17756569	98.75	16
F2_123	15494532	15315869	98.85	13.62
F2_124	14370932	14201670	98.82	13.12
F2_126	15726302	15544665	98.85	14.18
F2_127	16453738	16262323	98.84	14.64
F2_128	13428202	13270453	98.83	12.82
F2_130	12913072	12760715	98.82	12.71
F2_131	12862036	12718933	98.89	12.58
F2_133	14880624	14708380	98.84	13.88
F2_137	13676768	13491040	98.64	13.42
F2_138	14380168	14221232	98.89	13.24
F2_141	13949848	13792350	98.87	13.08
F2_143	14920652	14717967	98.64	14.61
F2_144	13948550	13786929	98.84	13.18
F2_145	14253206	14081716	98.8	13.29
F2_146	12493520	12342409	98.79	12.29
F2_147	10969184	10807146	98.52	11.88
F2_149	10964072	10807327	98.57	12.27
F2_150	11978440	11791740	98.44	12.64
F2_151	13175678	12996676	98.64	12.91
F2_152	12412392	12220160	98.45	12.55
F2_153	12103246	11931271	98.58	12.73
F2_156	11319242	11163982	98.63	12.27
F2_157	12134076	11968809	98.64	12.57
F2_158	11794724	11628422	98.59	12.42
F2_159	12170506	11974484	98.39	12.66
F2_160	11677792	11522751	98.67	12.34
F2_161	11551536	11404691	98.73	12.16
F2_162	10938238	10783673	98.59	12.17
F2_165	10587740	10445791	98.66	11.93
F2_166	10209280	10062457	98.56	12.13
F2_167	15147002	14935882	98.61	14.75
F2_169	14986492	14777299	98.6	14.97
F2_170	12550206	12379294	98.64	12.86
F2_173	12791724	12615515	98.62	13.82
F2_174	12339574	12159747	98.54	13.32

F2_176	13595620	13394254	98.52	13.9
F2_177	12865136	12700550	98.72	13.16
F2_178	13054716	12905291	98.86	14.4
F2_180	13036846	12896064	98.92	13.1
F2_182	15898140	15734679	98.97	14.04
F2_183	17686048	17496277	98.93	15.13
F2_186	15106582	14941902	98.91	13.72
F2_188	15424850	15253301	98.89	14.2
F2_190	15915964	15739796	98.89	14.24
F2_192	14273572	14106360	98.83	13.28
F2_194	14225140	14069665	98.91	13.49
F2_195	16910022	16720497	98.88	15.24
F2_196	11736452	11607345	98.9	12.47
F2_197	15761726	15590602	98.91	14.23
F2_198	13299862	13117734	98.63	12.93
F2_199	13804392	13642907	98.83	13.39
F2_200	15767222	15599509	98.94	14.09
F2_201	15817994	15659886	99	14.28
F2_202	16351964	16181096	98.96	14.28
F2_203	14775900	14619221	98.94	13.66
F2_205	15793502	15629433	98.96	14.3
F2_207	14180560	13990381	98.66	14.1
F2_211	16652042	16465299	98.88	15.2
F2_214	16116666	15910049	98.72	15.04
F2_215	13124670	12974673	98.86	12.41
Average	13876976	13694227	98.68	13.45

a: The totals of sequencing bases aligned to the 'Rbp' genome were divided by total genome bases in grasspea.
