# Supplementary material for: Construction of A GBS-Based High-Density Genetic Map and Flower Color-Related Loci Mapping in Grasspea (Lathyrus sativus L.)
Source: Plants (Basel). 2022 Aug 21;11(16):2172. doi: 10.3390/plants11162172 (PMC9414002; doi:10.3390/plants11162172)
Supplement: Supplementary file 1 [file plants-11-02172-s001.zip › Table S3.pdf]

Table S3. Statistic of homozygous SNP, heterozygous SNP, and Homozygous SNP rate in male (P1), female (P2), and F_2 individuals of grasspea.

Sample	Total	Homozygous SNP	Heterozygous SNP	Homozygous SNP rate (%)
P1_K714	551846	215557	336289	39.06
P2_IF1347	522270	319583	202687	61.19
F2_1	619922	274812	345110	44.33
F2_2	612207	288855	323352	47.18
F2_3	568896	276875	292021	48.67
F2_4	629634	331498	298136	52.65
F2_5	631052	324134	306918	51.36
F2_7	564857	270953	293904	47.97
F2_8	572366	255506	316860	44.64
F2_10	691193	346219	344974	50.09
F2_11	586184	264032	322152	45.04
F2_12	623978	317551	306427	50.89
F2_13	541591	261234	280357	48.23
F2_14	605938	275225	330713	45.42
F2_15	635364	323198	312166	50.87
F2_16	553633	267374	286259	48.29
F2_17	613478	309594	303884	50.47
F2_18	617410	308129	309281	49.91
F2_19	608151	276606	331545	45.48
F2_20	545225	296061	249164	54.3
F2_22	617833	297336	320497	48.13
F2_23	621798	309875	311923	49.84
F2_25	557347	277504	279843	49.79
F2_27	566418	285346	281072	50.38
F2_28	658585	306353	352232	46.52
F2_30	568140	271751	296389	47.83
F2_31	554684	274118	280566	49.42
F2_32	497735	222014	275721	44.6
F2_33	504255	223464	280791	44.32
F2_34	589437	262058	327379	44.46
F2_35	624058	280210	343848	44.9
F2_36	598631	284945	313686	47.6
F2_39	573161	274607	298554	47.91
F2_40	474121	211889	262232	44.69
F2_41	522262	249655	272607	47.8
F2_42	460739	209300	251439	45.43
F2_43	487210	206077	281133	42.3
F2_44	565612	278684	286928	49.27
F2_45	540137	241758	298379	44.76
F2_46	606803	264395	342408	43.57

F2_47	621982	277120	344862	44.55
F2_49	573915	275223	298692	47.96
F2_50	572768	264545	308223	46.19
F2_51	491037	211280	279757	43.03
F2_52	459361	218031	241330	47.46
F2_53	481407	213292	268115	44.31
F2_54	443276	196191	247085	44.26
F2_55	431622	211067	220555	48.9
F2_56	499019	215168	283851	43.12
F2_58	502996	242352	260644	48.18
F2_59	468190	210819	257371	45.03
F2_60	472770	204634	268136	43.28
F2_61	381360	161397	219963	42.32
F2_62	501536	247780	253756	49.4
F2_63	481818	238449	243369	49.49
F2_64	491776	222634	269142	45.27
F2_65	411158	200158	211000	48.68
F2_66	332676	145007	187669	43.59
F2_67	454881	223680	231201	49.17
F2_69	450389	202005	248384	44.85
F2_70	414846	194401	220445	46.86
F2_71	409800	201438	208362	49.16
F2_72	495664	245286	250378	49.49
F2_73	486182	208102	278080	42.8
F2_74	514582	245943	268639	47.79
F2_75	514913	288072	226841	55.95
F2_76	500149	242572	257577	48.5
F2_78	478864	241040	237824	50.34
F2_80	517183	263797	253386	51.01
F2_82	501133	262584	238549	52.4
F2_83	455663	231454	224209	50.79
F2_86	463700	197559	266141	42.6
F2_87	443590	219229	224361	49.42
F2_89	470035	234412	235623	49.87
F2_91	487363	215539	271824	44.23
F2_92	467808	225156	242652	48.13
F2_94	448969	200801	248168	44.72
F2_95	523796	222126	301670	42.41
F2_96	436651	204252	232399	46.78
F2_98	405463	202061	203402	49.83
F2_99	428753	191270	237483	44.61
F2_100	476096	204083	272013	42.87
F2_101	496764	235367	261397	47.38
F2_103	438879	194445	244434	44.3
F2_105	487147	214631	272516	44.06

F2_106	433158	224184	208974	51.76
F2_108	474452	199209	275243	41.99
F2_109	387484	169037	218447	43.62
F2_110	478680	233065	245615	48.69
F2_111	433705	183034	250671	42.2
F2_114	422571	182300	240271	43.14
F2_115	483708	217987	265721	45.07
F2_117	480494	235214	245280	48.95
F2_122	637009	312912	324097	49.12
F2_123	599514	264531	334983	44.12
F2_124	559695	215015	344680	38.42
F2_126	559969	267641	292328	47.8
F2_127	555657	297626	258031	53.56
F2_128	519574	258367	261207	49.73
F2_130	492377	235020	257357	47.73
F2_131	487616	204779	282837	42
F2_133	549222	257725	291497	46.93
F2_137	509230	270216	239014	53.06
F2_138	561488	244817	316671	43.6
F2_141	589719	243867	345852	41.35
F2_143	531715	253942	277773	47.76
F2_144	506446	242342	264104	47.85
F2_145	524600	230006	294594	43.84
F2_146	501840	202135	299705	40.28
F2_147	431320	185134	246186	42.92
F2_149	434280	216744	217536	49.91
F2_150	492569	239111	253458	48.54
F2_151	498508	244277	254231	49
F2_152	471080	204801	266279	43.47
F2_153	474406	184146	290260	38.82
F2_156	436936	214510	222426	49.09
F2_157	486506	209716	276790	43.11
F2_158	453265	191573	261692	42.27
F2_159	494293	237695	256598	48.09
F2_160	498457	214257	284200	42.98
F2_161	469192	211939	257253	45.17
F2_162	436533	214185	222348	49.07
F2_165	443399	193882	249517	43.73
F2_166	402433	195435	206998	48.56
F2_167	560527	263488	297039	47.01
F2_169	517031	240320	276711	46.48
F2_170	462035	224316	237719	48.55
F2_173	469190	217482	251708	46.35
F2_174	459682	238252	221430	51.83
F2_176	516728	234633	282095	45.41

F2_177	457440	214485	242955	46.89
F2_178	459694	225748	233946	49.11
F2_180	493056	244714	248342	49.63
F2_182	598527	267392	331135	44.68
F2_183	634051	333698	300353	52.63
F2_186	627011	281552	345459	44.9
F2_188	596454	260503	335951	43.68
F2_190	649376	284887	364489	43.87
F2_192	616443	254776	361667	41.33
F2_194	539860	256200	283660	47.46
F2_195	590534	297256	293278	50.34
F2_196	475121	221196	253925	46.56
F2_197	591535	276344	315191	46.72
F2_198	457232	219278	237954	47.96
F2_199	558771	287131	271640	51.39
F2_200	591856	310566	281290	52.47
F2_201	588427	323024	265403	54.9
F2_202	625132	304671	320461	48.74
F2_203	567418	250983	316435	44.23
F2_205	584741	274608	310133	46.96
F2_207	492117	256735	235382	52.17
F2_211	565473	291620	273853	51.57
F2_214	612745	294095	318650	48
F2_215	515650	205865	309785	39.92
Average	519799	244539	275260	46.97
