# Supplementary figures and images for: Construction of A GBS-Based High-Density Genetic Map and Flower Color-Related Loci Mapping in Grasspea (Lathyrus sativus L.)
Source: Plants (Basel). 2022 Aug 21;11(16):2172. doi: 10.3390/plants11162172 (PMC9414002; doi:10.3390/plants11162172)

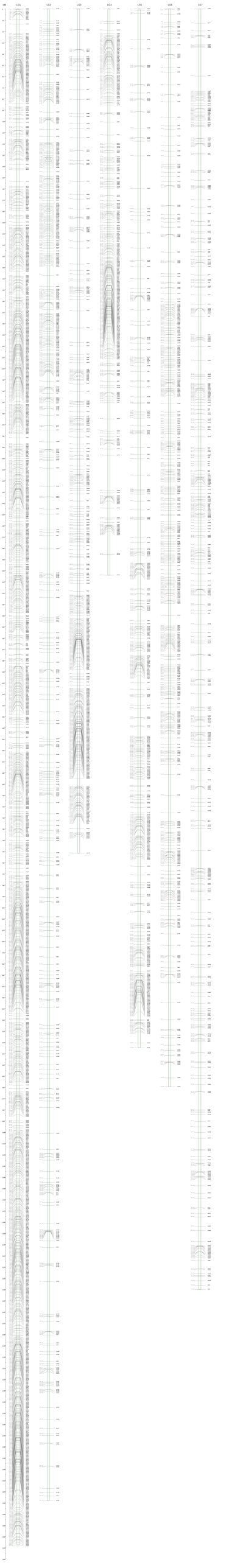

Supplement: Supplementary file 1 [file plants-11-02172-s001.zip › Figure S1.png]

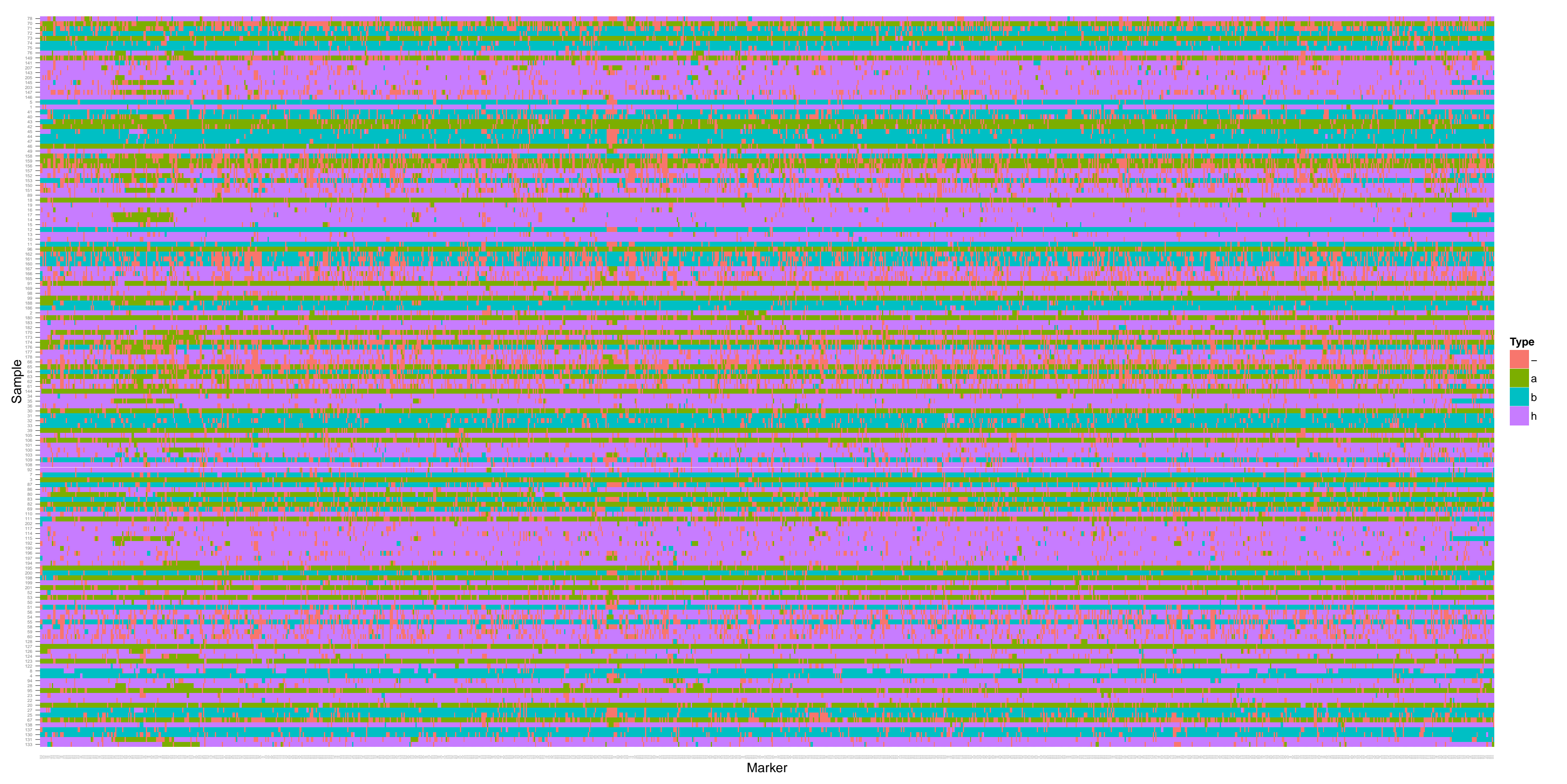

Supplement: Supplementary file 1 [file plants-11-02172-s001.zip › Figure S2-1 LG01.pdf]

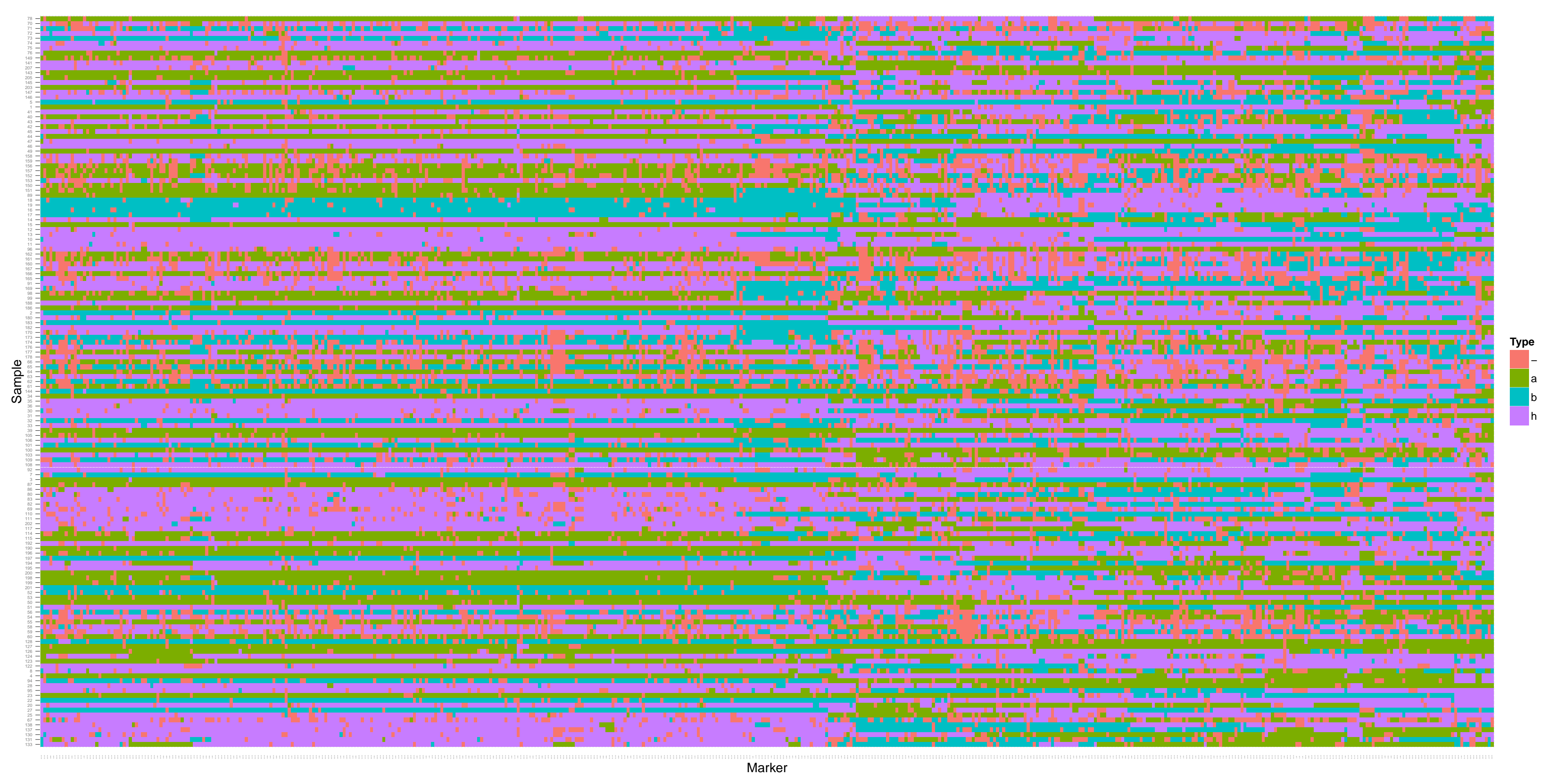

Supplement: Supplementary file 1 [file plants-11-02172-s001.zip › Figure S2-2 LG02.pdf]

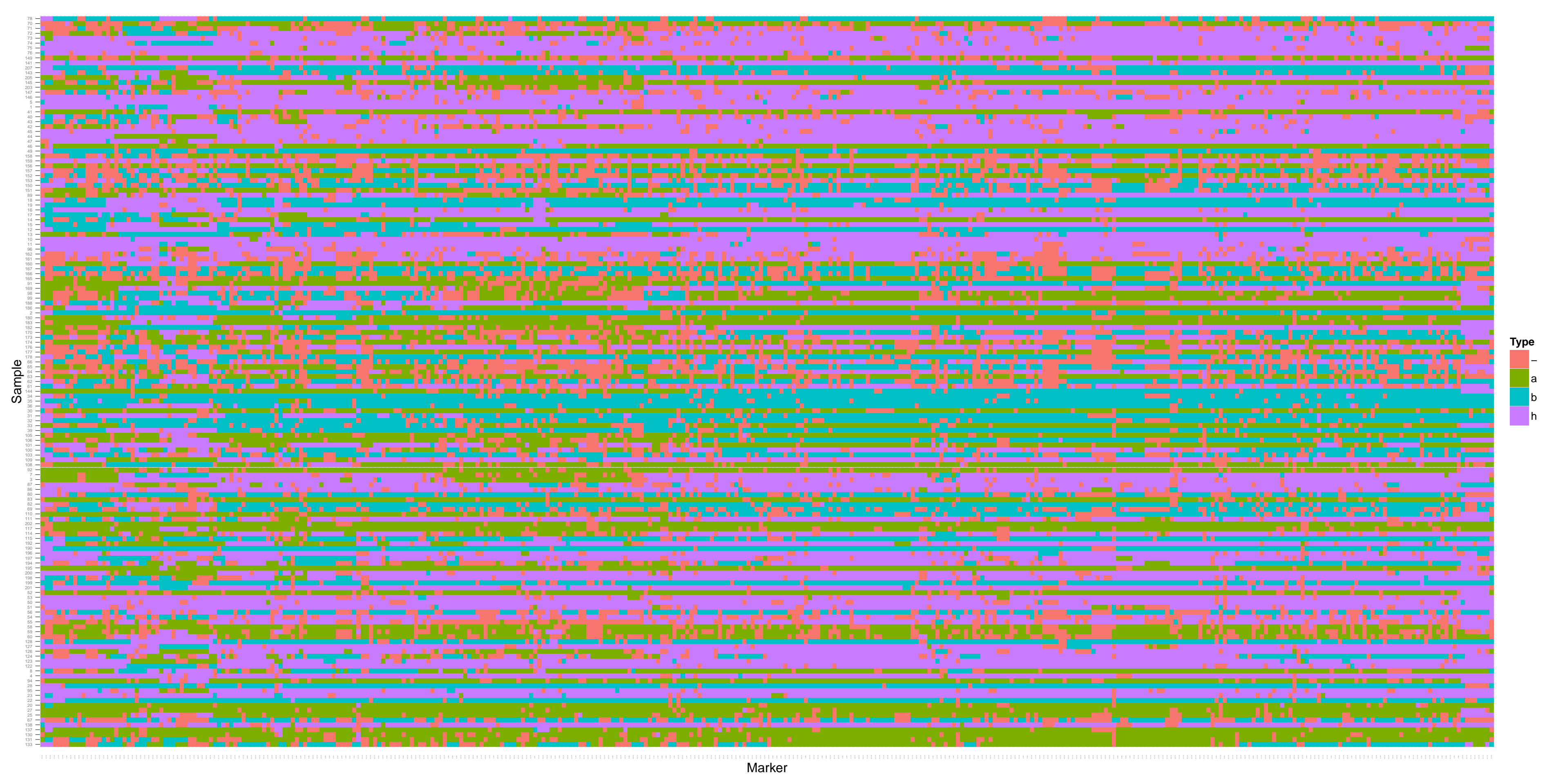

Supplement: Supplementary file 1 [file plants-11-02172-s001.zip › Figure S2-3 LG03.pdf]

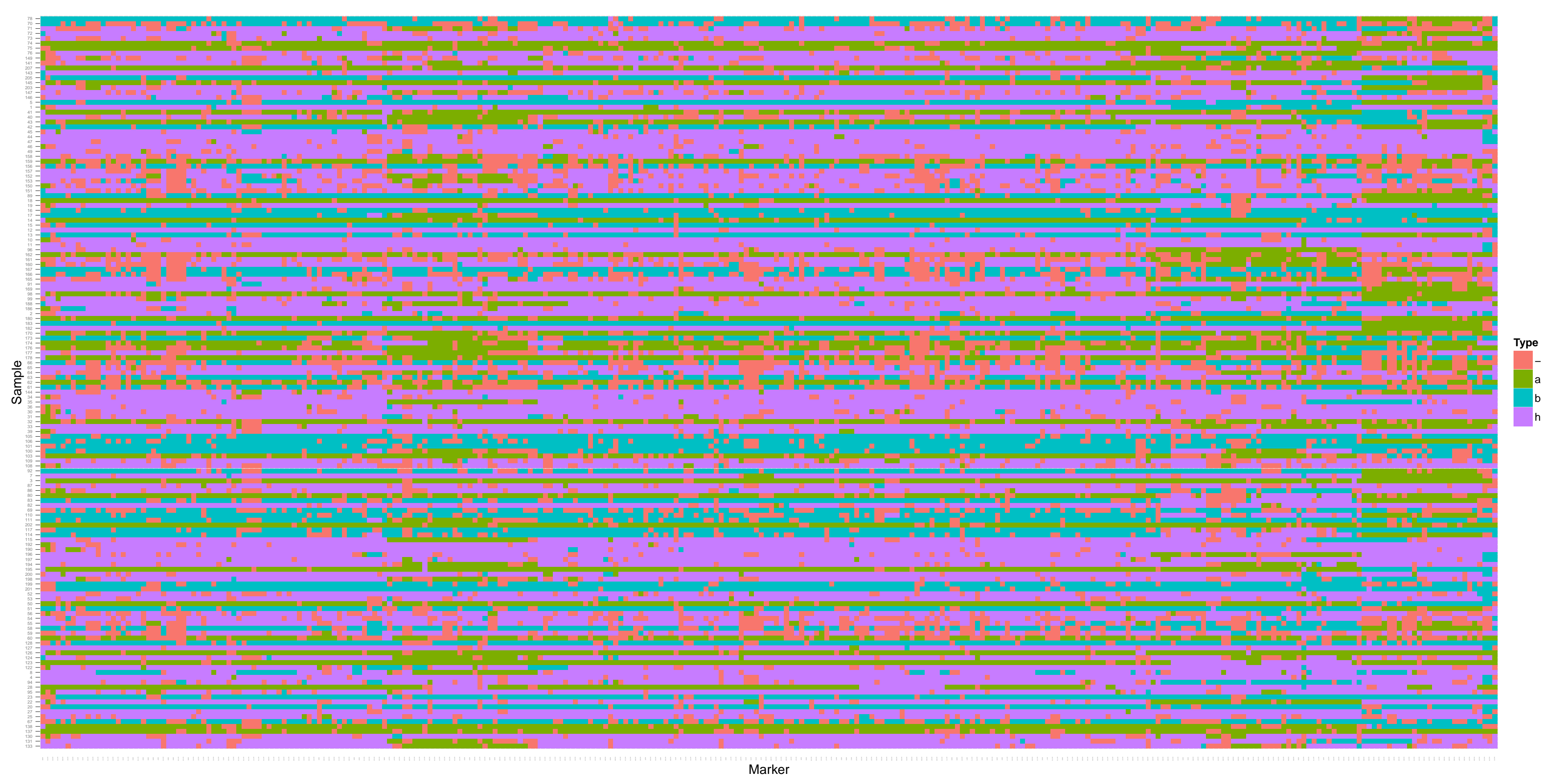

Supplement: Supplementary file 1 [file plants-11-02172-s001.zip › Figure S2-4 LG04.pdf]

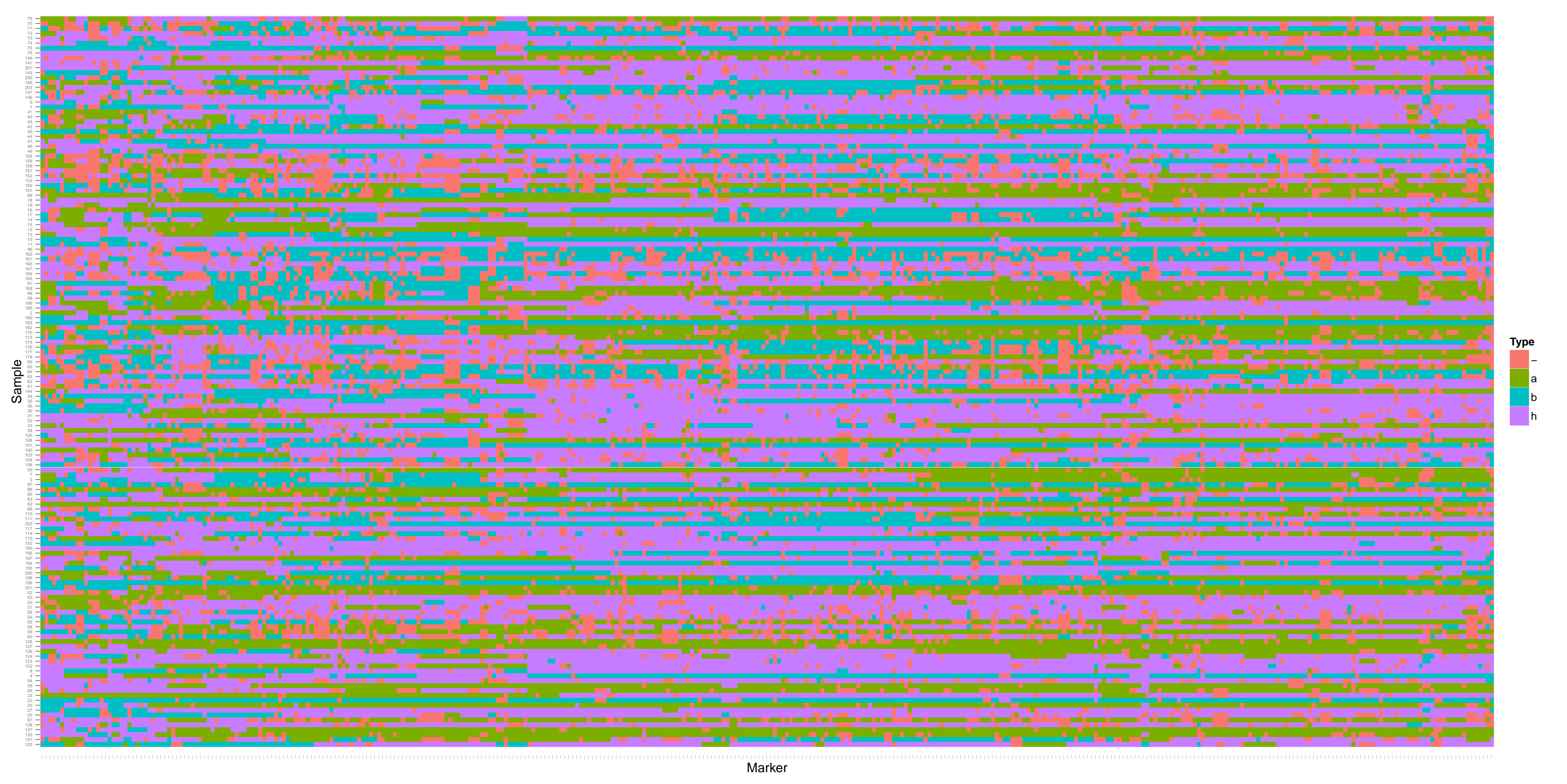

Supplement: Supplementary file 1 [file plants-11-02172-s001.zip › Figure S2-5 LG05.pdf]

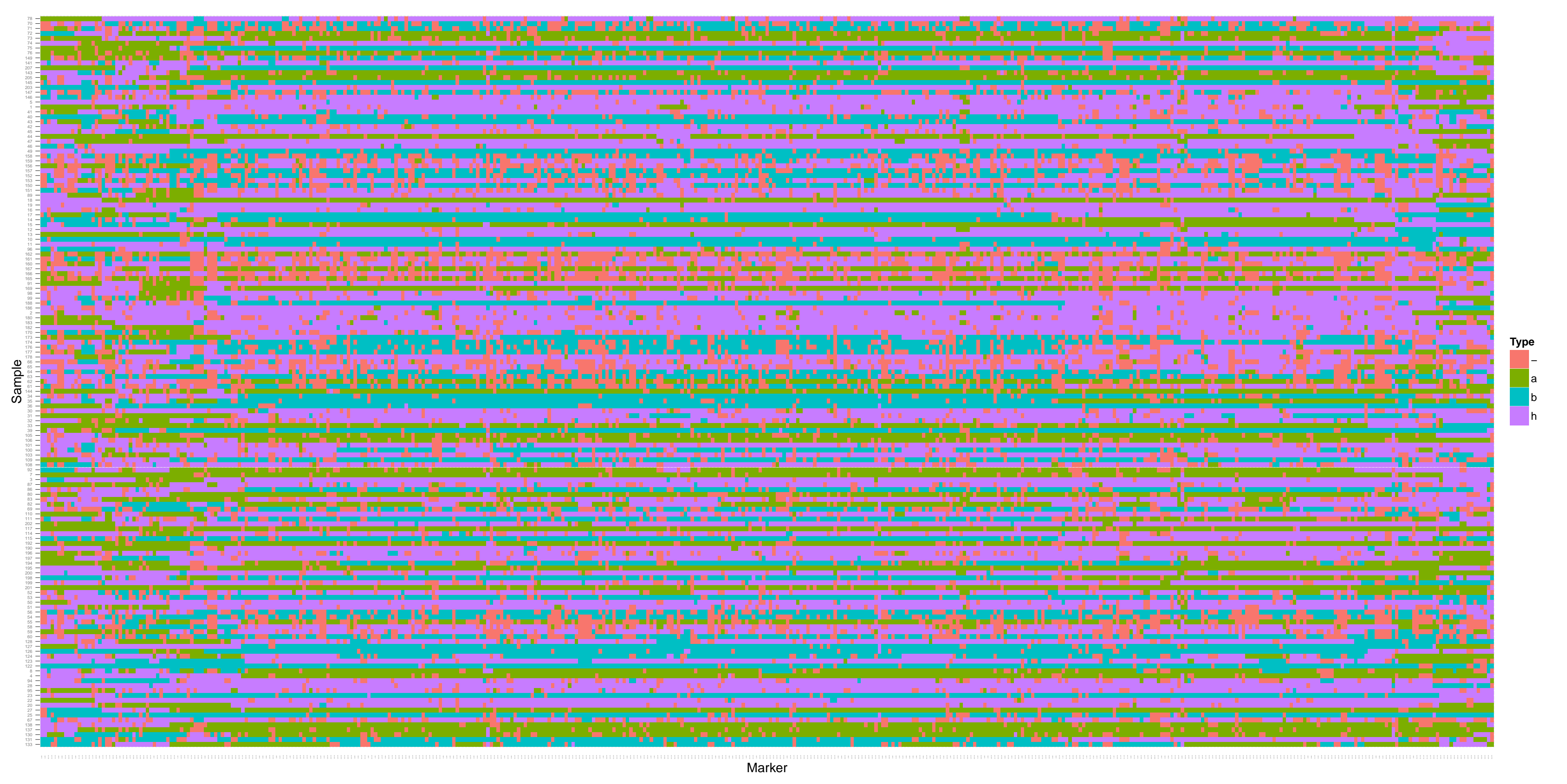

Supplement: Supplementary file 1 [file plants-11-02172-s001.zip › Figure S2-6 LG06.pdf]

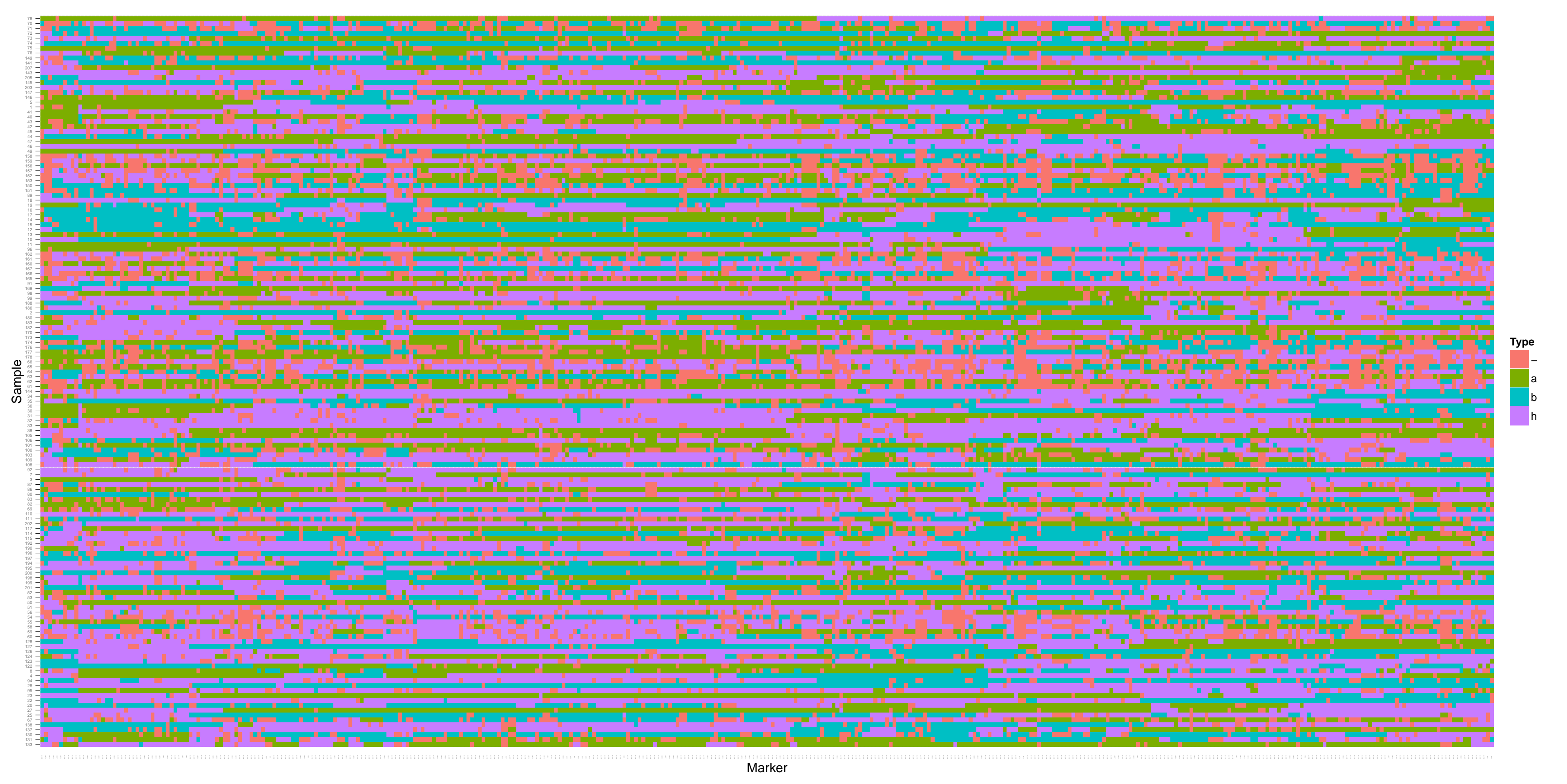

Supplement: Supplementary file 1 [file plants-11-02172-s001.zip › Figure S2-7 LG07.pdf]

lg01

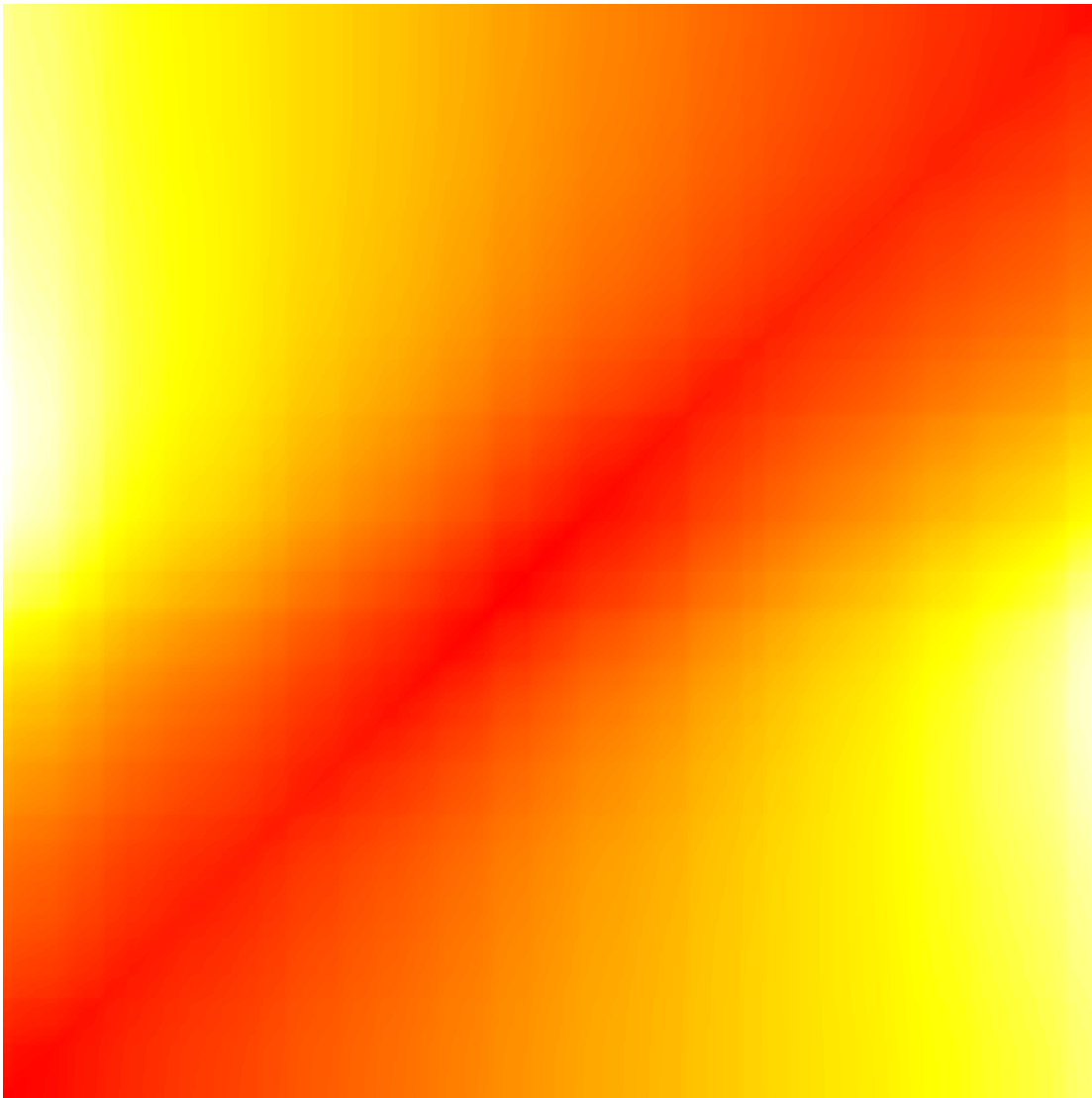

Supplement: Supplementary file 1 [file plants-11-02172-s001.zip › Figure S3-1 LG01.pdf]

Ig02

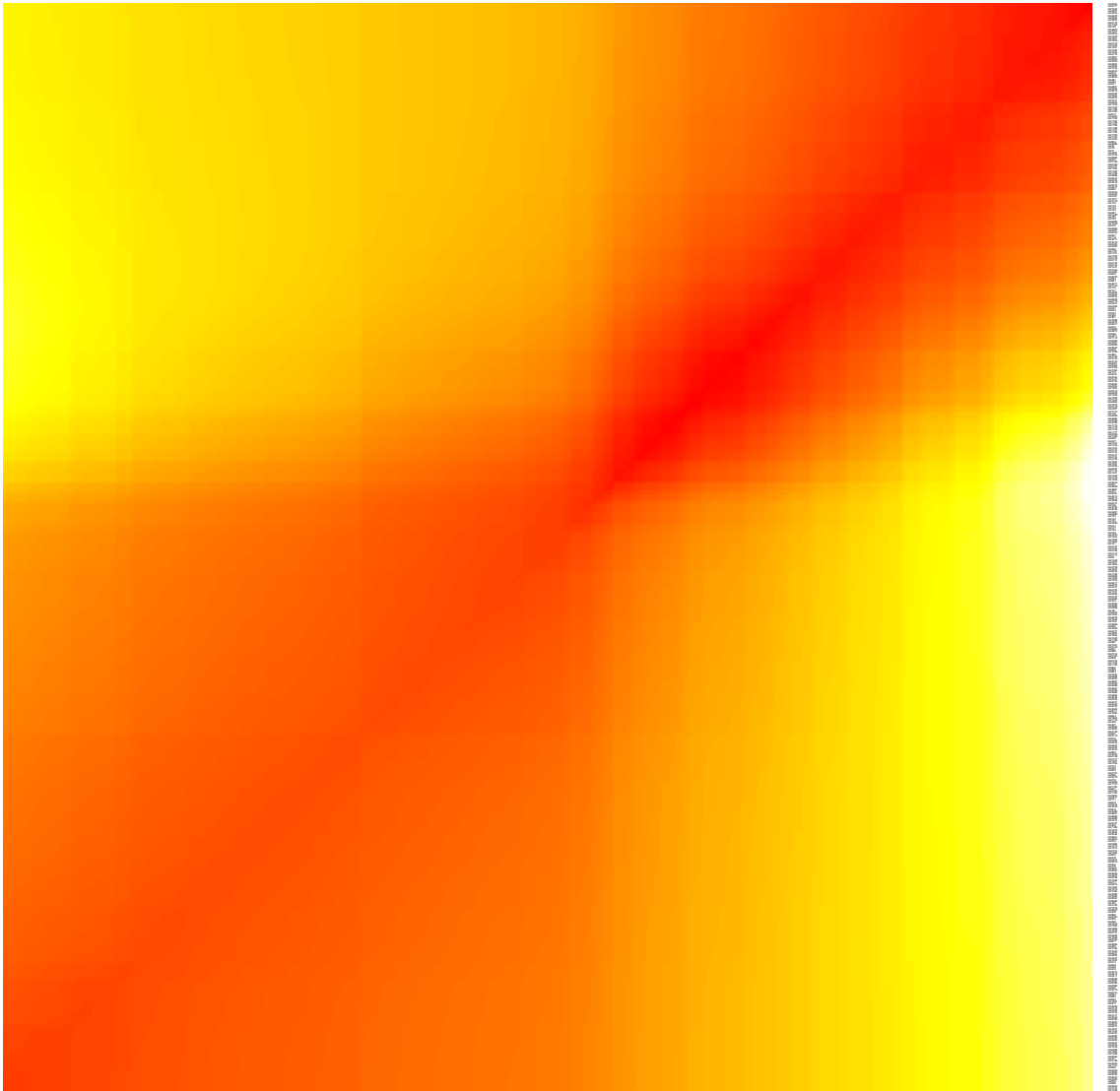

Supplement: Supplementary file 1 [file plants-11-02172-s001.zip › Figure S3-2 LG02.pdf]

lg03

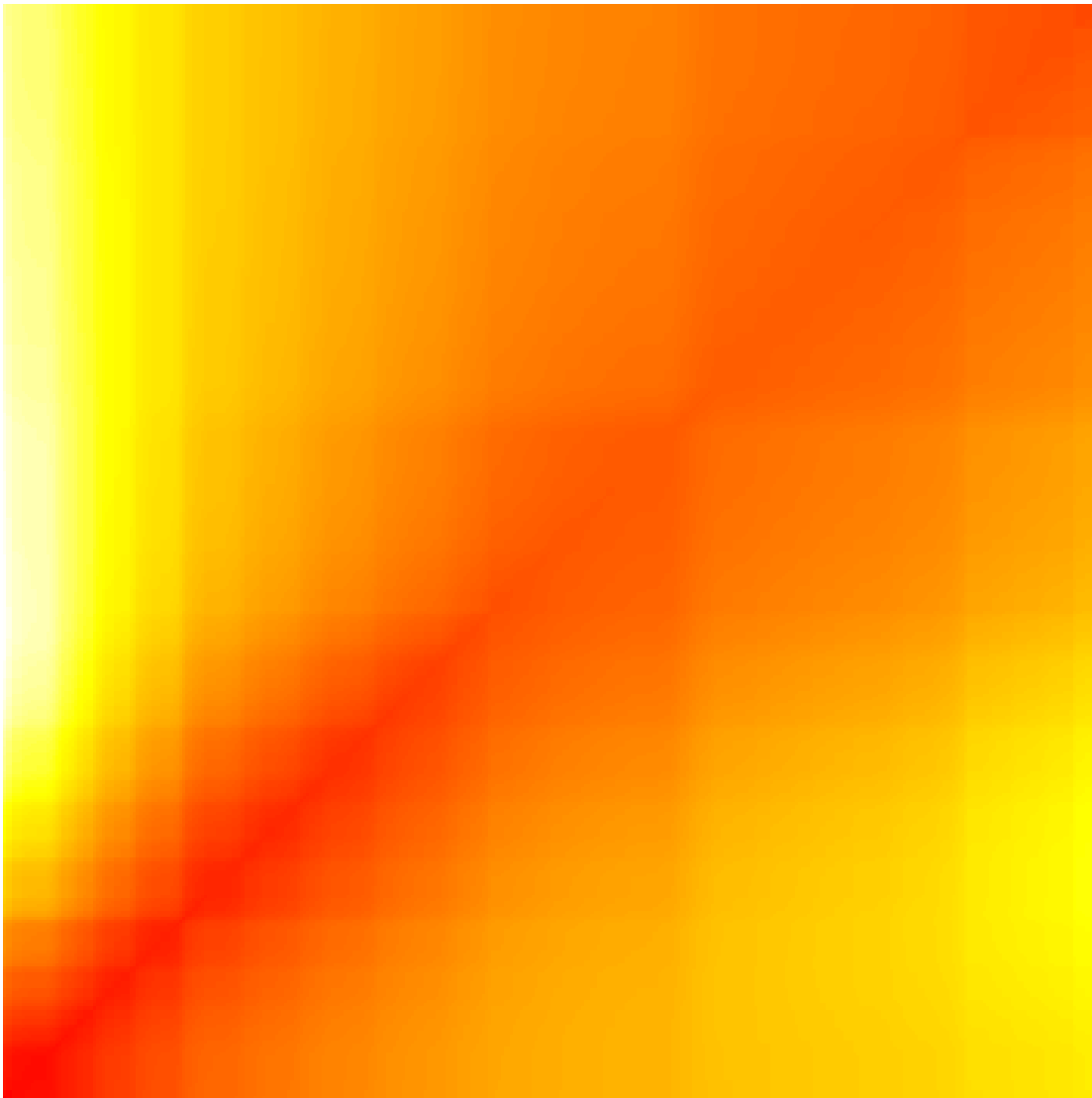

Supplement: Supplementary file 1 [file plants-11-02172-s001.zip › Figure S3-3 LG03.pdf]

lg04

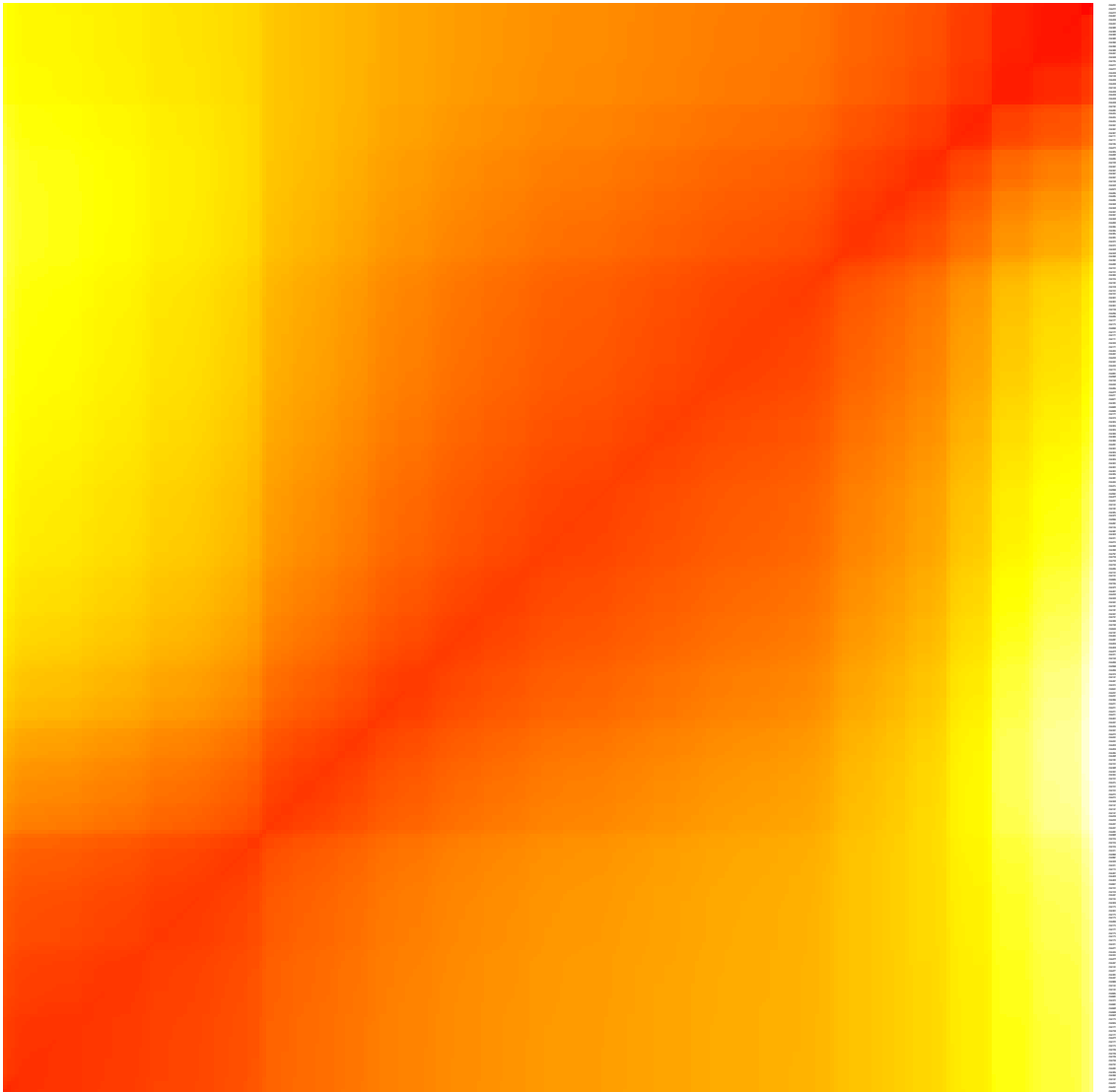

Supplement: Supplementary file 1 [file plants-11-02172-s001.zip › Figure S3-4 LG04.pdf]

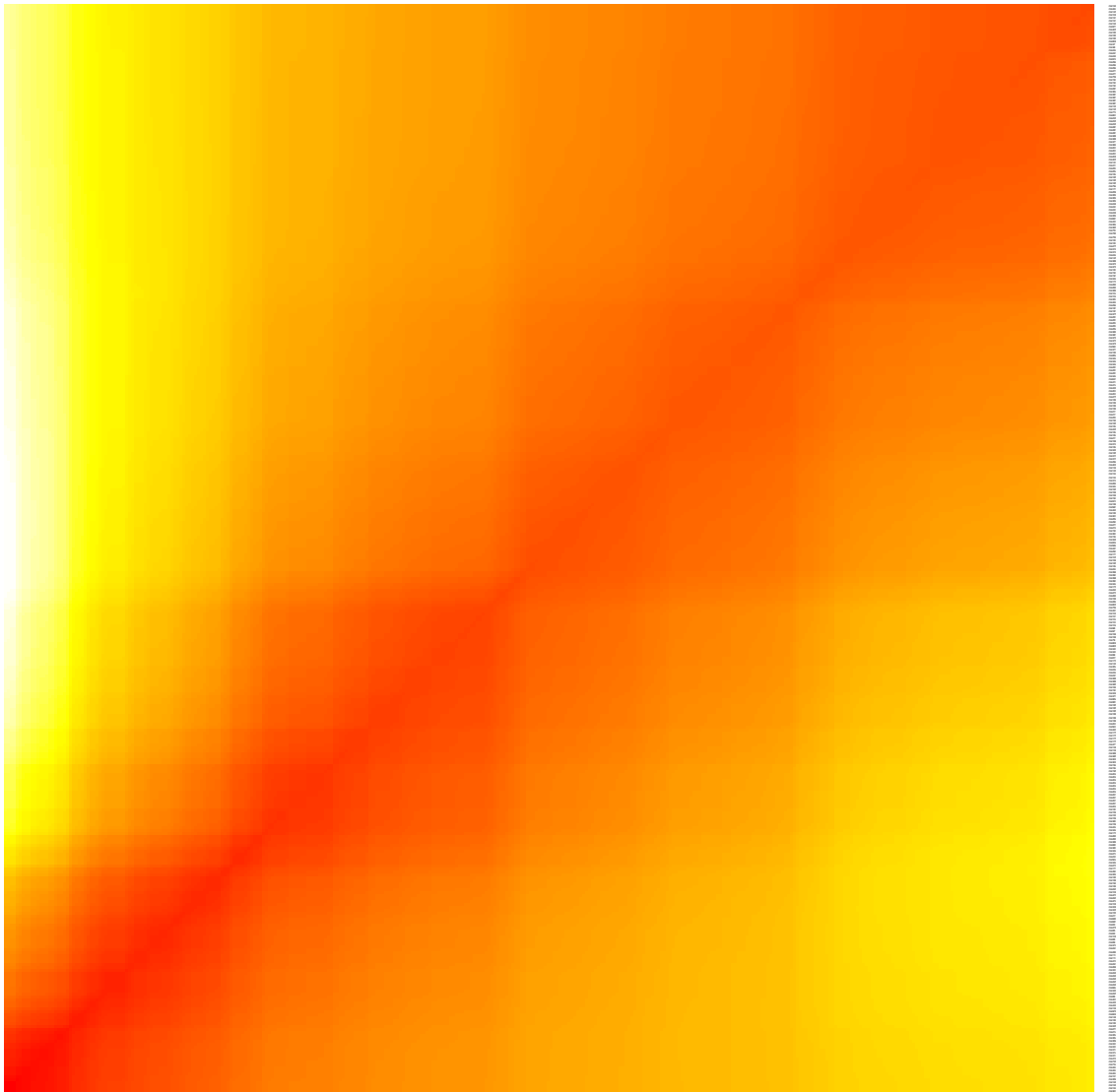

Supplement: Supplementary file 1 [file plants-11-02172-s001.zip › Figure S3-5 LG05.pdf]

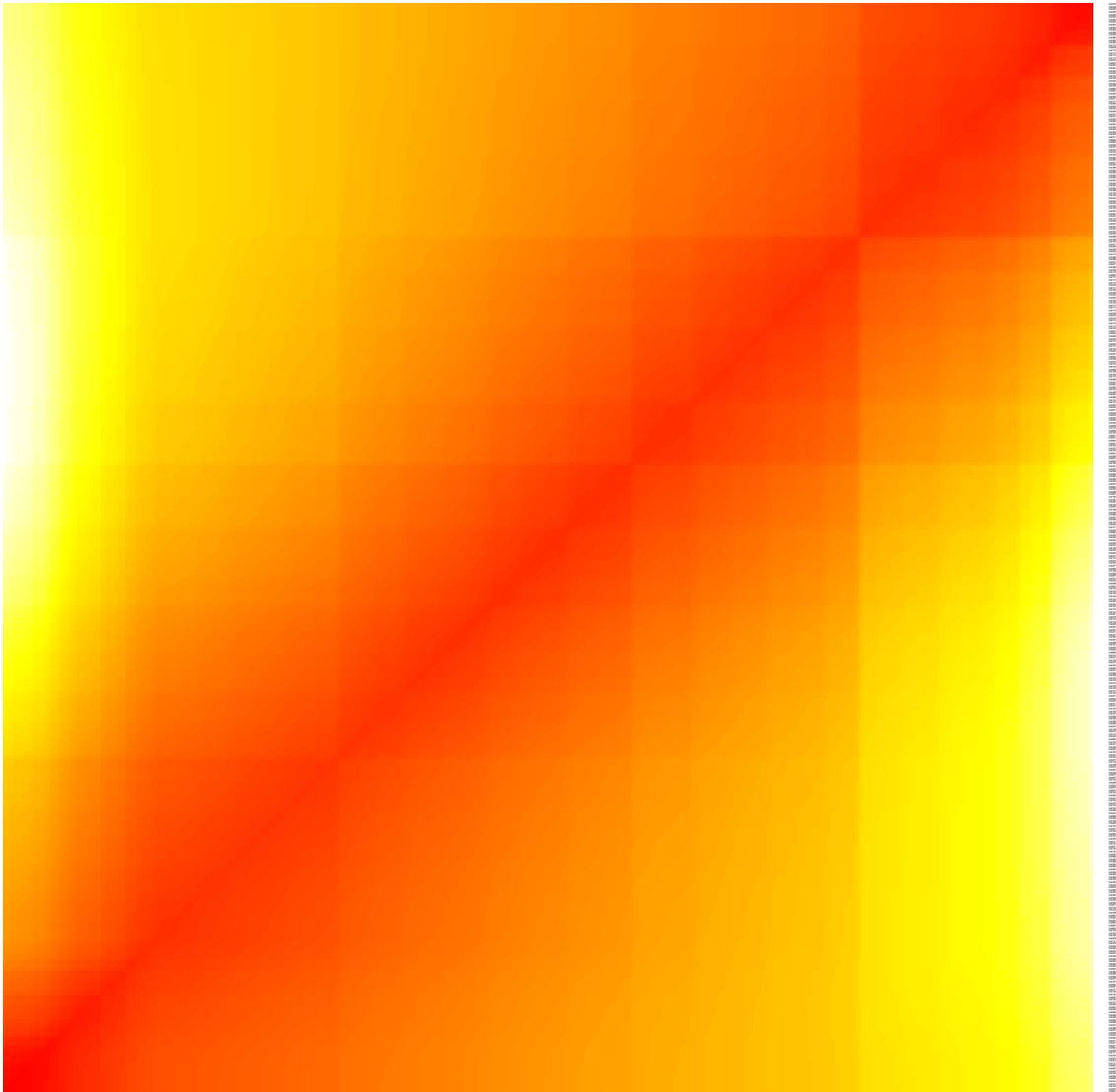

Supplement: Supplementary file 1 [file plants-11-02172-s001.zip › Figure S3-6 LG06.pdf]

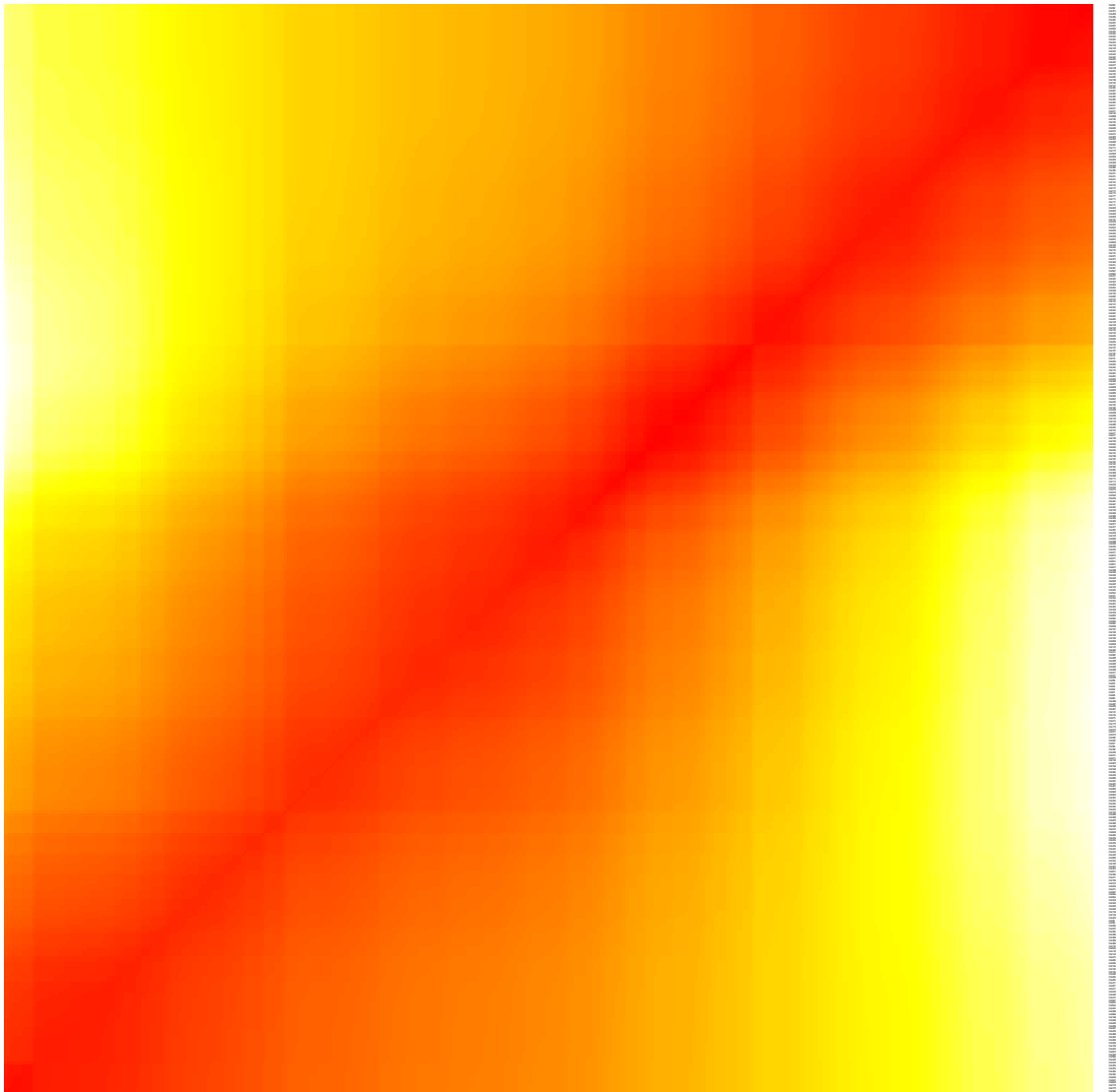

Supplement: Supplementary file 1 [file plants-11-02172-s001.zip › Figure S3-7 LG07.pdf]
